# Loss of YTHDC1 m^6^A *reading* function promotes invasiveness in urothelial carcinoma of the bladder

**DOI:** 10.1038/s12276-024-01377-x

**Published:** 2025-01-01

**Authors:** Jinyun Xu, Jonas Koch, Claudia Schmidt, Malin Nientiedt, Manuel Neuberger, Philipp Erben, Maurice Stephan Michel, Manuel Rodríguez-Paredes, Frank Lyko

**Affiliations:** 1 Division of Epigenetics, DKFZ-ZMBH Alliance, German Cancer Research Center, 69120 Heidelberg, Germany; 2 Core Facility Unit Light Microscopy, German Cancer Research Center, 69120 Heidelberg, Germany; 3 Department of Urology and Urosurgery, Medical Faculty Mannheim, University of Heidelberg, 68167 Mannheim, Germany

**Keywords:** Bladder cancer, RNA modification

## Abstract

Bladder cancer poses significant clinical challenges due to its high metastatic potential and poor prognosis, especially when it progresses to muscle-invasive stages. Here, we show that the m^6^A *reader* YTHDC1 is downregulated in muscle-invasive bladder cancer and is negatively correlated with the expression of epithelial‒mesenchymal transition genes. The functional inhibition or depletion of YTHDC1 increased the migration and invasion of urothelial cells. Integrative analysis of multimodal sequencing datasets provided detailed insights into the molecular mechanisms mediating YTHDC1-dependent phenotypes and identified SMAD6 as a key transcript involved in the invasiveness of urothelial carcinoma of the bladder. Notably, SMAD6 mRNA colocalized less with YTHDC1 in tumoral tissues than in paratumoral tissues, indicating disrupted binding during cancer progression. Our findings establish YTHDC1-dependent m^6^A reading as a critical epitranscriptomic mechanism regulating bladder cancer invasiveness and provide a paradigm for the epitranscriptomic deregulation of cancer-associated networks.

## YTHDC1 loss drives invasiveness in bladder cancer

Bladder cancer is a common and potentially deadly disease affecting the urinary system. Researchers have identified a protein called YTHDC1, which reads specific RNA modifications (chemical changes to RNA that affect its function), as being less present in more aggressive cases of bladder cancer. This study investigates how YTHDC1 influences the invasiveness of cancer cells. The research involved analyzing bladder cancer samples and conducting experiments on cell models, using RNA sequencing (a method to study the sequence of RNA) and other techniques to understand YTHDC1’s role in cancer progression. Results indicated that lower levels of YTHDC1 are associated with increased cancer cell invasion and migration. The study concludes that YTHDC1 is crucial in preventing cancer spread by regulating specific genes. Future research could explore YTHDC1 as a potential target for bladder cancer treatment.

This summary was initially drafted using artificial intelligence, then revised and fact-checked by the author.

## Introduction

Bladder cancer (BLCA) is a potentially lethal cancer of the urinary system that ranks as the fourth most common cancer among men^1^. In 30% of patients with the main subtype, urothelial carcinoma of the bladder (UCB), the tumors reach the muscles of the organ, a stage called muscle-invasive bladder cancer (MIBC), which is linked to a worse prognosis owing to greater metastatic potential^2^. The five-year overall survival rate for BLCA patients decreases to 8% in patients with distant metastases^1^, emphasizing the critical need to fully understand the molecular mechanisms that facilitate the spread of cancer cells in this type of tumor.

Epithelial‒mesenchymal transition (EMT) is a biological process in which epithelial cells undergo dynamic changes to acquire mesenchymal characteristics, leading to increased migration capacity and invasiveness^3^. Recent evidence suggests that EMT is not a simple conversion between two distinct states but rather represents a spectrum of transitional phases known as partial or hybrid EMT^4,5^. The transition of BLCA cells to a mesenchymal state is well documented and is associated with tumor progression, metastasis, and resistance to chemotherapy^6,7^.

N^6^-methyladenosine (m^6^A) is the most common internal mRNA modification in eukaryotes and is catalyzed by the m^6^A *writer* complex^8–11^. This modification can regulate gene expression through RNA splicing, RNA stability, export, and translation^12^. The m^6^A *writer* methyltransferase-like 3 (*METTL3*) is upregulated in BLCA, contributes to tumor proliferation and invasion, and is associated with poor survival^13–15^. However, the specific function of m^6^A modification in BLCA is still poorly understood. Indeed, our recent findings suggest a rather complex role of METTL3 in UCB, including increased expression but lower global m^6^A mRNA levels than in paratumoral tissue^16^.

Different m^6^A *readers* recognize modified sites and function as mediators of various regulatory mechanisms. YT521-B homology (YTH) domain-containing proteins, including YTHDF1-3, YTHDC1, and YTHDC2, are the main m^6^A *readers* and specifically recognize modifications with their evolutionarily conserved YTH domain^10^. Some of these proteins have also been implicated in tumorigenesis, including that of UCB. An interesting example is YTHDF2, which has recently been shown to facilitate UCB progression by inhibiting the RIG-I-mediated immune response^17^. Another example is YTHDC1, which is located mainly in the nucleus and modulates pre-mRNA splicing, mRNA export and mRNA stabilization in HEK293T and HeLa cells^18–20^. YTHDC1 has been linked to various physiological processes and to the development of different tumor types. In ovarian cancer, for example, YTHDC1 has been found to act as a tumor suppressor in a m^6^A-dependent manner through its binding and stabilization of *PIK3R1* mRNA^21^. In clear cell renal cell carcinoma (ccRCC), *YTHDC1* expression was found to be reduced and associated with poor prognosis^22^. Finally, in BLCA, *YTHDC1* downregulation has recently been shown to promote tumorigenesis specifically under hyperglycemic conditions and in a m^6^A-dependent manner, which was linked to increased glycolysis and mediated by the glucose transporter GLUT3^23^. In these tumors, decreased levels of YTHDC1 are also associated with cisplatin resistance through m^6^A-dependent destabilization of *PTEN* mRNA^24^.

In this study, we aimed to explore the specific role of YTHDC1 in the invasiveness of UCB, a crucial process for the outcome of this disease that has not yet been systematically investigated. The results from our own cohort of UCB patients revealed lower *YTHDC1* mRNA and protein expression levels in more invasive MIBC cases, along with a negative correlation in the expression of key EMT genes. Consistently, treating urothelial cell models with a YTHDC1 inhibitor increased their invasion and migration, similar to that of YTHDC1-depleted cells. Integrated analysis of subsequent RNA sequencing (RNA-seq) and RNA immunoprecipitation sequencing (RIP-seq) experiments allowed us to identify *SMAD6* as a key transcript with a role in UCB invasion that is dependent on YTHDC1 m^6^A *reader* function.

## Materials and Methods

### Patients and patient samples

For our study of *YTHDC1* expression, we used a cohort of UCB patients from the Department of Urology and Urosurgery at Mannheim University Hospital that we had previously described (see Supplementary Table 1 for clinicopathological details^16^), as well as data from the UROMOL (European Genome-phenome Archive, accession EGAS0000100469319) and the Fudan (see Supplementary Table 1 for clinicopathological details) cohorts^25,26^. To explore the subsequent impact of *YTHDC1* levels on patient survival, we also used a cohort of UCB patients from Mannheim University Hospital. Patients with a follow-up time of ≤ 60 days were excluded to avoid bias due to perioperative complications.

IHC and RNA FISH assays were performed on uropathologist-diagnosed, formalin-fixed, paraffin-embedded (FFPE) UCB samples prepared according to standard protocols (see Supplementary Table 2 for clinicopathological details)^27^. All of these tumors were characterized according to the TNM classification for BLCA by the Union for International Cancer Control (UICC 2017), excluding those with histopathological findings other than UCB. This study was conducted in accordance with the Declaration of Helsinki, and all patients provided consent to participate. Additionally, approval was obtained from Ethics Committee II of the University of Heidelberg (2013-845R-MA).

### Cell culture

All of the cell lines used in this study were first authenticated by single nucleotide polymorphism profiling, tested for mycoplasma contamination, and cultured according to the ATCC guidelines. Thus, the healthy urothelial cell line UROtsa, which was originally derived from a 12-year-old female, was cultured in RPMI 1640 medium supplemented with 5% fetal bovine serum (FBS) and 1% penicillin/streptomycin (P/S). The BLCA cell lines UM-UC-3 (derived from a male of unspecified age), T24 (derived from an 82-year-old female), RT112 (derived from a 63-year-old male), and RT4 (derived from a 63-year-old male) were used in this study. HEK293T (derived from a female, age unspecified), UM-UC-3 and RT112 cells were cultured in high-glucose DMEM supplemented with 10% FBS and 1% P/S, whereas T24 and RT4 cells were maintained in McCoy’s 5 A (modified) medium supplemented with 10% FBS and 1% P/S. All of the cell lines were maintained as adherent monolayers at 37 °C in a humidified incubator with a 5% CO_2_ atmosphere.

### Plasmid constructs and lentiviral infection

CRISPR/Cas9-mediated depletion of YTHDC1 was achieved using LentiCRISPRv2GFP, which was a gift from David Feldser (Addgene plasmid # 82416)^28^. In this case, the sequence of the sgRNA used to target *YTHDC1* was 5’-GGATGAGAGAGAGAGACCAGAAAG-3’, whereas that of the nontargeting control sgRNA was 5’-GCTGACGGCGAGCTTTAGGC-3’. For shRNA-mediated YTHDC1 depletion, the plasmid pLVX-shRNA2 (Clontech) was used. Here, the shRNA sequence targeting *YTHDC1* was 5’-GGAGGAAGAGATGAAGAAGAAGTA-3’, whereas the control sequence was 5’-GGACGAAGATGATGATGAAGTA-3’. For YTHDC1 overexpression, the coding sequence (CDS) was amplified via PCR from the cDNA of WT UROtsa cells and subsequently cloned and inserted into a modified pN251 plasmid, which was originally a gift from Le Cong (Addgene plasmid #188471)^29^. For SMAD6 overexpression, the CDS was PCR amplified from the cDNA of WT UROtsa cells with an HA tag and subsequently cloned and inserted into the pLVX-IRES-tdTomato (Clontech) vector. For lentiviral production, these plasmids were cotransfected with Lipofectamine 2000 (Thermo Fisher Scientific) into HEK293T cells together with psPAX2 and pMD2.G vectors. psPAX2 was a gift from Didier Trono (Addgene plasmid #12260), and pMD2.G was a gift from Didier Trono (Addgene plasmid # 12259). After incubation for 48 h, the virus-enriched media were centrifuged and filtered with 0.42 μm cellulose acetate filters. Subsequently, UROtsa cells were exposed to these media for 48 h and then fluorescence-activated cell sorting (FACS)-sorted according to the highest GFP/tdTomato signal (top 20%). This ensured that only homogeneous populations of transduced cells highly expressing the constructs of interest were obtained. For *SMAD6* knockdown, small interfering RNAs (siRNAs) (Silencer™ Select, Thermo Fisher Scientific) were used, and *Silencer*™ Select Negative Control No. 1 siRNA (Thermo Fisher Scientific, #4390843) was purchased. The sequence of the siRNA targeting *SMAD6* was 5’-ACAAAAAGCUAAUACCAGUtt-3’. Transfection was performed using Lipofectamine 2000 (Invitrogen), where 250 pmol siRNA was used to transfect 150,000 UROtsa cells, and the cells were harvested after 24–72 h.

### In vitro cell-based assays

Cell viability was assessed via a CellTiter-Glo kit (Promega) following the manufacturer’s instructions. For UROtsa cells, 1500 cells per well were seeded in 96-well plates, and measurements were performed for five days at 24 h intervals. For all BLCA cell lines, the cells were seeded in 96-well plates and treated with 1 μg/mL doxycycline to induce YTHDC1 expression after attachment. Measurements from 24 to 96 h were normalized with respect to the reference measurement made at 0 hours. For the clonogenic assays, 1000 UROtsa cells per well were seeded in 6-well plates. BLCA cell lines were seeded at 500 cells per well in 6-well plates and induced with 1 μg/ml doxycycline after attachment. After 10 days, the cells were fixed with ice-cold methanol for 10 min and subsequently stained with 0.05% crystal violet at room temperature for 10 minutes. Colony formation was quantified via the ColonyArea plugin of ImageJ^30^. To assess apoptosis, UROtsa cells were also seeded in 96-well plates at a density of 10,000 cells per well, and caspase 3/7 activity was measured 24 hours later using the Caspase-Glo 3/7 Assay Kit (Promega). For the BLCA cell lines, 8000 cells per well were seeded in 96-well plates and induced with 1 μg/mL doxycycline after attachment. Caspase 3/7 activity was measured 24 h after induction. To normalize the number of seeded cells, Cell Titer-Glo viability measurements were performed in parallel, as described above.

Finally, to assess cell migration and invasion, the BLCA cell lines were first seeded in 6-well plates and induced with 1 μg/mL doxycycline after attachment. Eight-micrometer polyester (PET) membrane Transwell inserts were placed in 24-well plates (Corning, #3464). For the invasion assays, these inserts were precoated with 1.2 mg/mL Matrigel basement membrane matrix (Corning, #354234) diluted in serum-free medium (1:8 dilution), which was allowed to solidify in an incubator for 3 h. Then, the cells were detached, centrifuged and resuspended in serum-free medium at a concentration of 10^5^ cells/mL. A 200 µL aliquot of the cell suspension was then seeded onto each insert, and 600 µL of medium containing 10% FBS was added to the bottom of each well. After 24 h of incubation at 37 °C, the cells that did not migrate or invade were removed from the insert with a cotton swab, while the cells that had migrated or invaded were fixed with 4% paraformaldehyde (PFA) prior to their staining with 0.1% crystal violet. For quantification, five random fields were counted at 200X magnification and imaged with an optical microscope.

### RT-qPCR

Regarding the FFPE tumor sections from UCB patients, RNA isolation was carried out from a single 10 μm thick section, after the identification of the tumor regions of each sample, using a XTRAKT FFPE Kit (Stratifyer, Köln, Germany). From the cell lines, RNA was isolated via the TRIzol method (Thermo Fisher Scientific) according to the manufacturer’s instructions. cDNA synthesis was subsequently carried out in all samples via the SuperScript III First-Strand Synthesis System (Thermo Fisher Scientific), and the Mesa Green qPCR MasterMix Plus assay for SYBR (Eurogentec, Seraing, Belgium) was used for real-time quantitative PCR. The specific primers used for these assays were as follows: for *YTHDC1*, forward 5’-TCCTTCACAGATGGGTTCTGTC-3’ and reverse 5’-GATGCAGAGCTTCCACTTCTATC-3’; for *CALM2*, forward 5’-GAGCGAGCTGAGTGGTTGTG-3’ and reverse 5’-AGTCAGTTGGTCAGCCATGCT-3’; for *SMAD6*, forward 5’- CACTGAAACGGAGGCTACCAAC-3’ and reverse 5’- CCTGGTCGTACACCGCATAGAG-3’; and for *RPS23*, forward 5’-TGGAGGTGCTTCTCATGCAA-3’ and reverse 5’-AATGGCAGAATTTGGCTGTTTG-3’. The expression levels were finally determined in triplicate for each sample to ensure reproducibility and accuracy. The 2^−ΔΔCt^ method was used for analysis of the qPCR data, and normalization was performed using the housekeeping genes *CALM2* and *RPS23*.

### Western blotting

Cell lysates were prepared using urea buffer (8 M urea, Tris-HCl pH 8.0). Proteins were then separated by SDS‒PAGE and transferred to nitrocellulose membranes using a Trans-Blot Turbo Transfer System (Bio-Rad). After the membranes were subsequently blocked with 5% milk in PBS-T at room temperature for 1 h, primary antibody incubation was performed overnight at 4 °C with anti-YTHDC1 (Proteintech, #29441-1-AP, 1:1000 dilution), anti-HA (Roche, # 11867423001, 1:1000 dilution), anti-histone-H3 (Proteintech, #17168-1-AP, 1:2000 dilution) and anti-β-actin (Santa Cruz Biotechnology, #sc-47778, 1:1000 dilution) antibodies. The incubation with the appropriate secondary antibodies was also performed at room temperature for 1 h. Images were finally acquired with the chemiluminescent HRP Immobilon Western HRP Substrate (Merck Millipore) and an M6 ECL ChemoStar device (Intas) prior to analysis with the ChemoStar V60+ and ImageJ software packages.

### Immunohistochemistry

Slides with FFPE tissue sections were first deparaffinized and rehydrated, followed by antigen retrieval via microwave heating. The sections were then blocked with FBS and incubated overnight with an anti-YTHDC1 primary antibody (#29441-1-AP, Proteintech) at a 1:500 dilution. After incubation for one additional hour with an anti-rabbit HRP secondary antibody at room temperature, images were acquired using an AxioScan 7 slide scanner (Zeiss) with a 10x objective. The quantification was evaluated with the Immunoreactive Score (IRS), which was calculated by multiplying scores for the distribution (0: 0%, 1: <10%, 2: 10–50%, 3: 51–80%, 4: > 80%) and intensity (0 = no staining, 1 = mild staining, 2 = moderate staining, 3 = intense staining) of the immunostaining^31^.

### RNA sequencing (RNA-seq)

Total RNA was extracted from cells via TRIzol reagent (Thermo Fisher Scientific) following the manufacturer’s protocol. The samples were then digested with DNAse I to remove potential genomic DNA contamination and cleaned with an RNA Clean & Concentrator Kit (Zymo Research). After library generation with the Illumina TruSeq Stranded RNA Kit following the manufacturer’s protocol, the cDNA concentration was measured using the Qubit dsDNA HS Assay Kit (Life Technologies), whereas cDNA integrity was checked using D1000 ScreenTape (Agilent Technologies). Paired-end sequencing (100 bp) was finally performed on a NovaSeq 6000 system (Illumina) with S4 flow cells.

For the analysis, raw RNA-seq data quality was first evaluated via FastQC (version 0.11.9). The adaptor sequences were subsequently trimmed via Trimmomatic (version 0.33) prior to the application of any data filtering criteria. The reads were then aligned to the human reference genome (GRCh38.p13 assembly) using HISAT2 (version 2.2.1), and the mapped reads were quantified using featureCounts (version 2.0.6)^32^. The data were finally normalized geometrically, and differential gene expression analysis was conducted using DEseq2 (version 1.42.0)^33^ with |Log_2_FoldChange | > 1.5 and *q* < 0.05. Gene set enrichment analysis (GSEA) was conducted with GSEA software^34^ and gene sets from the Molecular Signatures Database (MSigDB) (version 2023.1.Hs)^35^. Gene Ontology (GO) analyses were performed with shinyGO (version 0.77)^36^, using the differentially expressed genes (DEGs) that complied with |Log_2_FoldChange | ≥1.5, *q* < 0.05.

### RNA immunoprecipitation sequencing (RIP-seq) and RIP-qPCR

For RIP-seq, UROtsa wild-type cells were treated with either DMSO or 50 μM METTL3 inhibitor STM2457 and harvested for RNA immunoprecipitation after 24 h. RNA immunoprecipitation was performed in 2 biological replicates for each group via the Magna RIP RNA-binding Protein Immunoprecipitation Kit (Millipore) following the manufacturer’s guidelines. Whole-cell lysates were prepared by a freeze‒thaw cycle and then precleared by incubation with IgG antibodies and beads consecutively for 30 min each. After centrifugation, 1/10 of the supernatant was stored as input samples, and the remaining supernatant was then incubated overnight with protein A/G magnetic beads and 5 μg of anti-YTHDC1 primary antibody (#29441-1-AP, Proteintech). The pulled-down protein was removed with Proteinase K, and the coprecipitated RNA was isolated via phenol:chloroform:isoamyl alcohol (125:24:1). For subsequent sequencing, RNA libraries were prepared using the SmarTer Ultra Low Input RNA v4 and NEBNext ChIP-Seq kits following the manufacturer’s protocols and then subjected to paired-end (100 bp) RNA-seq performed on a NovaSeq 6000 sequencing system (Illumina) via SP flow cells. RIP‒qPCR was performed to validate the binding between YTHDC1 and *SMAD6* and the blockade of the YTHDC1 inhibitor. Thus, UROtsa wild-type cells were treated with either DMSO or 50 μM of the inhibitor for 6 h before being harvested for RNA immunoprecipitation. RNA immunoprecipitation was performed as described above. The cell extracts were incubated overnight with protein A/G magnetic beads and 5 μg of anti-YTHDC1 (#29441-1-AP, Proteintech) or negative control IgG antibody. The isolated RNA was then cleaned with an RNA Clean & Concentrator Kit (Zymo Research) and subsequently analyzed via RT‒qPCR.

For the RIP-seq analysis, raw reads were initially trimmed using TrimGalore (version 0.6.10) and mapped to rRNA (sourced from the National Center for Biotechnology Information Nucleotide database) using Bowtie2 (version 2.5.2). Unmapped reads were then aligned to the human genome and transcriptome (hg38 annotation) using HISAT2 (version 2.2.1) and the GENCODE annotation files (version 21, https://gtexportal.org/home/datasets), and duplicate reads and those with low mapping quality were filtered out using the filterdup function from MACS2 (version 2.2.9.1). Peak calling was performed via MACS2 (version 2.2.9.1) with --nomodel and --extsize 200 parameters, and peaks with a *q*-value < 0.01 were considered significant. Significant peaks identified in both biological replicates were then merged via BEDtools (version 2.31.1) and used in downstream analyses. In this context, RIP peak annotation and motif identification were carried out via the annotatePeaks.pl and findMotifsGenome.pl scripts from Homer (version 4.11). For visualization purposes, the BAM files corresponding to the immunoprecipitated and input samples were converted to Bigwig format, and the read coverage was normalized to the input library size via RPKM normalization using deeptools (version 3.5.4). Differentially bound sites were identified using Diffbind (version 3.6.1) with default parameters. To compare the signal distribution in DMSO- and STM2457-treated cells, immunoprecipitated samples were normalized to their corresponding inputs and their library size using deeptools’ bamCompare (version 3.5.4). The normalized peaks were finally visualized using coolbox (version 0.3.8) with the corresponding genome tracks.

### RNA FISH and IF codetection

All of the FFPE samples were sectioned into consecutive 5 μm thick slices for RNA FISH and IF analyses. Each slide was stained with hematoxylin and eosin (H&E) and digitized using an AxioScan 7 slide scanner (Zeiss) with a 20x objective. To simultaneously detect YTHDC1 protein and *SMAD6* RNA signals, we used the RNAscope® Multiplex Fluorescent Reagent Kit v2 (#323285, ACDbio) together with the RNA‒Protein Co-Detection Ancillary Kit (#323285, ACDbio). Probes specific to the human *SMAD6* transcript were obtained from ACDbio (#900421). Briefly, FFPE sections from all samples were deparaffinized with xylene and 100% ethanol before being subjected to a target retrieval step. The sections were then incubated with primary YTHDC1 antibody (#29441-1-AP, Proteintech, dilution: 1:200) overnight at 4 °C. Protease treatment was subsequently applied to all of the samples, and the probes were then hybridized with the tissue samples. Following several signal amplification steps, the fluorophores were coupled to the probes. After two washes, the sections were incubated with the secondary antibody (goat anti-rabbit Alexa Fluor 750 #A-21039, Invitrogen, dilution: 1:200) at room temperature for 2 h. Finally, the sections were counterstained with DAPI and mounted with ProLong Gold Antifade Mountant (#P36930, Thermo Fisher). Images were acquired via an AxioScan 7 (Zeiss) slide scanner microscope with a 40X objective. To detect YTHDC1 and *SMAD6* signals and their colocalization, we used the Big-FISH package (version 0.6.2)^37^. The main steps included denoising the image and detecting the local maximum in the filtered image, after which the bright and dense areas were decomposed. Colocalization was quantified as the percentage of colocalized *SMAD6* foci, which was calculated by dividing the number of colocalized *SMAD6* spots by the total number of *SMAD6* spots. To determine the number of spots per cell, we utilized CellPose (version 3.0.5)^38^ to count nuclei using the DAPI signal.

### Dual luciferase assays

The wild-type 5’UTR of *SMAD6* was PCR amplified from wild-type UROtsa cells, and the m^6^A-site-mutated (A to C) 5’UTR of *SMAD6* was synthesized by GeneArt (Thermo Fisher). Both fragments were then cloned upstream of the firefly luciferase gene in pFL-SV40. pFL-SV40 was a gift from Ming-Chih Lai (Addgene plasmid # 115352)^39^. The wild-type and m^6^A-mutant *SMAD6* 5’UTR firefly luciferase pFL-SV40 plasmids were each cotransfected with the Renilla luciferase pIS1 vector (pIS1 was a gift from David Bartel (Addgene plasmid # 12179)^40^) into UROtsa Ctrl and YTHDC1-depleted cells. After 48 h, the activities of both firefly and Renilla luciferases were measured using the Dual-Glo Luciferase Assay Kit (#E2920, Promega) according to the manufacturer’s protocol.

### Statistical analysis

All of the cellular experiments were performed in 3 biological replicates, and each experiment was conducted in triplicate, unless otherwise specified. The data were analyzed using the statistical environments provided by Python. For comparisons between two groups, unpaired two-tailed Student’s t tests were performed. When three or more groups were compared, one-way analysis of variance (ANOVA) followed by Tukey’s post hoc test for multiple comparisons was used, unless otherwise specified. In all analyses, significance levels were defined as follows: not significant (ns) *p*-value ≥ 0.05, *p-value < 0.05, **p-value < 0.01, and ***p-value < 0.001. The graphs and error bars represent the means ± standard deviations (SDs), unless otherwise specified. Survival analyses were performed using the Kaplan‒Meier method, and the results were compared using the log-rank test. Image analyses were performed with the FIJI and Qupath software.

## Results

### YTHDC1 downregulation is associated with the metastatic potential of UCB

**Fig. 1:**
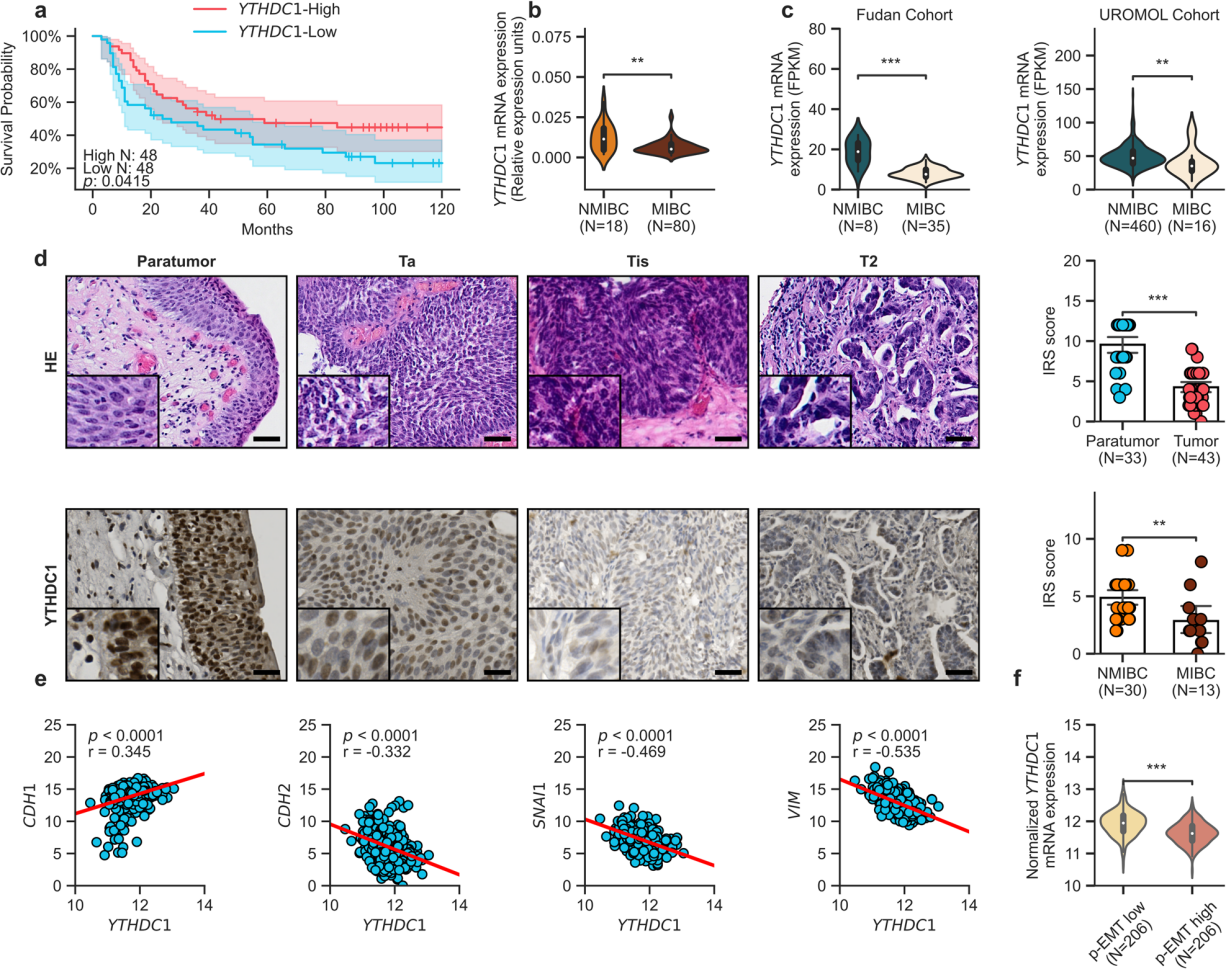
YTHDC1 is downregulated and associated with metastatic potential in urothelial carcinoma of the bladder. **a** Kaplan‒Meier ten-year overall survival analysis of patients with different levels of *YTHDC1* using data from the Mannheim University Hospital cohort. Statistical significance was assessed by the log-rank test. **b** Comparison of *YTHDC1* mRNA expression between NMIBC (*n* = 18) and MIBC (*n* = 80) patients in the Mannheim University Hospital cohort. **p-value < 0.01, Mann‒Whitney U test. **c** *YTHDC1* mRNA expression levels in NMIBC compared with MIBC samples from the published Fudan cohort and UROMOL cohorts. ***p-value < 0.001, **p-value < 0.01, Mann‒Whitney U test. **d** Representative H&E staining (upper panel) and IHC (lower panel) results showing YTHDC1 expression across different stages of bladder cancer. Scale bar = 100 μm. Quantitative analyses of the YTHDC1 IHC assays are shown in the right panels, which were performed via the IRS method and compared between paratumoral and tumor tissues (above), as well as between NMIBC and MIBC tissues (below). ***p-value < 0.001, **p-value < 0.01, Mann‒Whitney U test. **e** Pearson correlation analyses between *YTHDC1* expression (Log_2_(normalized counts +1)) and canonical EMT markers (Log_2_(normalized counts +1)) in the TCGA-BLCA dataset, with *p*-values and correlation coefficients (r) provided. **f** Relative *YTHDC1* expression (Log_2_(normalized counts +1)) in the p-EMT high (*n* = 206) and p-EMT low (*n* = 206) groups in the TCGA-BLCA dataset. ***p value < 0.001.

Recently, lower *YTHDC1* levels in the tumors of BLCA patients were shown to confer a worse outcome^23^. However, this finding was based on the clinically limited TCGA dataset, which does not permit the highly relevant differentiation between nonmuscle invasive bladder cancer (NMIBC) and MIBC. For a more detailed analysis of YTHDC1 in the clinical progression of UCB, we analyzed *YTHDC1* mRNA levels in a suitable subset of patients (see Supplementary Table 1 for details) selected from our previously described cohort^16^. In agreement with published findings^23^, a ten-year overall survival analysis of this cohort revealed that patients with lower *YTHDC1* expression had a worse prognosis (Fig. 1a). Similarly, MIBC cases, which are more prone to metastasize^2^, presented lower *YTHDC1* mRNA levels than NMIBC cases (Fig. 1b). This result was further confirmed (Fig. 1c) using two other recently published^25,26^ datasets (UROMOL and Fudan cohorts; see Supplementary Table 1 for details). Furthermore, immunohistochemistry (IHC) analysis of tissue sections revealed lower YTHDC1 expression in UCB than in paratumoral tissue, which is consistent with previous analyses^23,24^. Importantly, the invasive and more advanced T2 stage cases presented significantly (*p*-value < 0.01, Mann‒Whitney U test) lower levels of YTHDC1 protein expression than did the nonmuscle invasive Ta and Tis stages (Fig. 1d and Supplementary Fig. 1a). No significant differences were found between the Ta and Tis cases (Supplementary Fig. 1a).

Since the metastatic potential of MIBC is closely related to EMT^41^, we next investigated the expression of EMT-related genes in the TCGA-BLCA dataset. Indeed, *YTHDC1* expression was negatively correlated with mesenchymal markers, such as *CDH2*, *SNAI1*, *SNAI2*, *ZEB1*, *VIM* and *PRRX1*^42^, whereas epithelial markers, such as *CDH1*, *EPCAM*, *OVOL1* and *OVOL2*^43^, were positively correlated (Fig. 1e and Supplementary Fig. 1b). Finally, we investigated whether *YTHDC1* is associated with a partial epithelial‒mesenchymal transition (p-EMT) state^5^. Interestingly, TCGA-BLCA cases can be classified into previously described p-EMT high and low subgroups^5^, with *YTHDC1* expression being significantly lower in the p-EMT high subgroup than in the p-EMT low subgroup (Fig. 1f). Taken together, these results are consistent with a role of *YTHDC1* downregulation in promoting the invasiveness of UCB tumors.

### Inhibition of YTHDC1 induces invasion and migration in urothelial cells

**Fig. 2:**
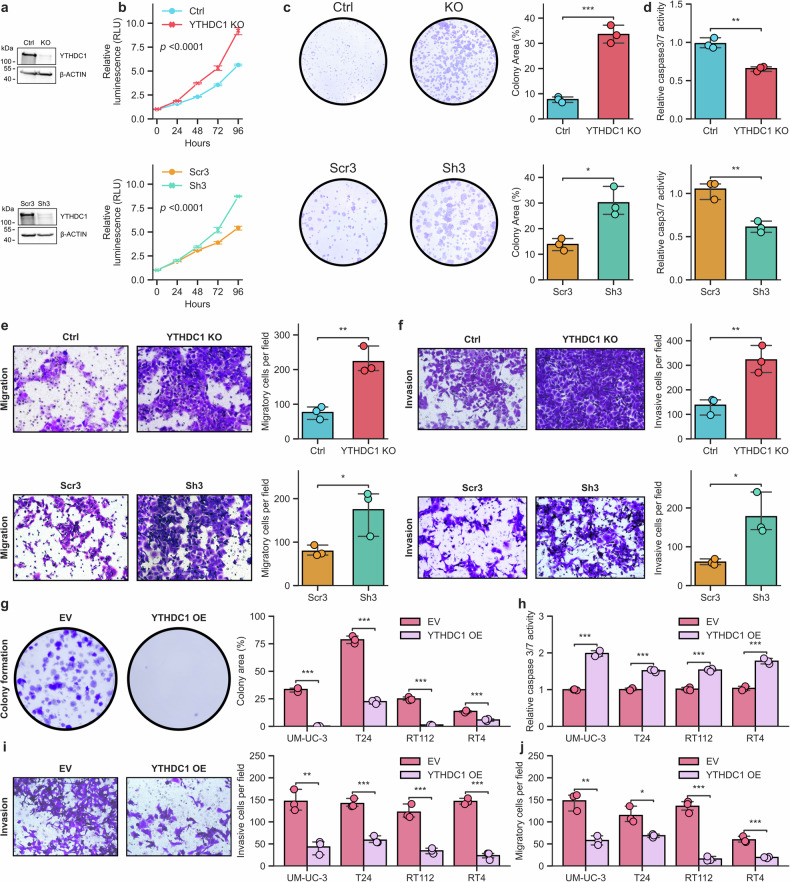
Cancer-promoting effects of YTHDC1 depletion in urothelial cells. **a** Western blot showing YTHDC1 depletion (upper panel) and knockdown (lower panel) in UROtsa cells. **b** Viability of UROtsa cells upon YTHDC1 depletion (upper panel) or knockdown (lower panel), as analyzed by the CellTiter-Glo assay. The experiments were performed in biological and technical triplicates. *p*-value < 0.0001, two-way analysis of variance (ANOVA). **c** Colony formation assays in UROtsa YTHDC1 control (Ctrl) and YTHDC1-depleted (KO) cells (above) or UROtsa control (Scr3) and YTHDC1-knockdown (Sh3) cells (below). Representative images are displayed on the left, while the quantification and statistics of three independent replicates using the ColonyArea algorithm in ImageJ are shown on the right. ***p-value < 0.001, *p-value < 0.05; unpaired two-sided t test. **d** Apoptotic cell levels in UROtsa cells depleted of YTHDC1 compared with those in control (above) and sh3 vs. Scr3 cells (below), as measured by Caspase-3/7-Glo assays with biological and technical triplicates. **p-value < 0.01, unpaired two-sided *t* test**. e** Transwell migration assays for YTHDC1 Ctrl and YTHDC1-depleted (upper panel) or Scr3 and Sh3 (lower panel) UROtsa cells. Representative images were taken using a 20x objective lens and are displayed on the left, while the quantification and statistics of the relative number of migrated cells are shown on the right. **p-value < 0.01, *p-value < 0.05, unpaired two-sided *t* test. **f** Transwell invasion assays for YTHDC1 Ctrl and YTHDC1-depleted (above), or Scr3 and Sh3 (below) UROtsa cells. Representative images were taken using a 20x objective lens and are displayed on the left, while the quantification and statistics of relative cell invasion are shown on the right. **p-value < 0.01, *p-value < 0.05, unpaired two-sided *t* test. All Transwell experiments were performed with 3 biological replicates. **g** Colony formation assays in BLCA cell lines (UM-UC-3, T24, RT112, and RT4) with the YTHDC1 empty vector (EV) or YTHDC1-overexpressing (OE) cells. Representative images of UM-UC-3 cells are displayed on the left, while quantification and statistics of all BLCA cell lines from three independent replicates using the ColonyArea algorithm in ImageJ are shown on the right. ***p-value < 0.001, unpaired two-sided t test. **h** Apoptosis levels in BLCA cell lines with YTHDC1 overexpression compared with those with empty vector, as measured by Caspase-3/7-Glo assays with biological and technical triplicates. ***p-value < 0.001, unpaired two-sided *t* test. **i** Transwell invasion assays in BLCA cell lines with YTHDC1 EV and YTHDC1 OE. The representative images shown are the invasion assays in UM-UC-3 cell lines, which were taken with a 20x objective lens and are displayed on the left. Representative images of UM-UC-3 cells (left, 20x objective) and quantification of all of the cell lines (right) are shown. ***p-value < 0.001, **p-value < 0.01, unpaired two-sided *t* test. **j** Quantification of migration assays in BLCA cell lines with empty vector or YTHDC1 overexpression. ***p-value < 0.001, **p-value < 0.01, unpaired two-sided *t* test.

To further investigate whether the decrease in *YTHDC1* expression observed in UCB tumors is related to increased invasiveness, we used the healthy urothelial cell model UROtsa to generate cells with reduced *YTHDC1* expression via short hairpin RNAs and CRISPR-Cas9. The reduction was confirmed by both Western blot and qRT‒PCR (Fig. 2a and Supplementary Fig. 2a), with residual expression levels likely due to *YTHDC1* gene essentiality (Supplementary Fig. 2b). Phenotypic assays performed with YTHDC1-depleted cells revealed significantly increased viability (KO and sh3: *p*-value < 0.0001, two-way analysis of variance) and clonogenicity (KO: *p*-value < 0.001, sh3: *p*-value < 0.05, two-tailed Student’s *t* test) of urothelial cells (Fig. 2b, c). Moreover, lower levels of YTHDC1 also decreased the apoptotic capacity of UROtsa cells (KO and sh3: *p*-value < 0.01, two-tailed Student’s *t* test), as evidenced by the lower activity of caspases 3 and 7 (Fig. 2d). To investigate the effect of YTHDC1 reduction on cell migration and invasion, we performed Transwell assays, which revealed a significantly (KO: *p*-value < 0.01, sh3: *p*-value < 0.05, two-tailed Student’s *t* test) greater capacity of the YTHDC1-depleted cells to migrate and invade (Fis. 2e, f). Conversely, the overexpression of YTHDC1 (Supplementary Fig. 2c) in several UCB cell lines (UM-UC-3, T24, RT112 and RT4), which represent various subtypes of UCB^44–46^, consistently led to suppressed cell proliferation, migration and invasion while promoting apoptosis across all UCB cell lines (Fig. 2g–j and Supplementary Fig. 2d–f).

**Fig. 3:**
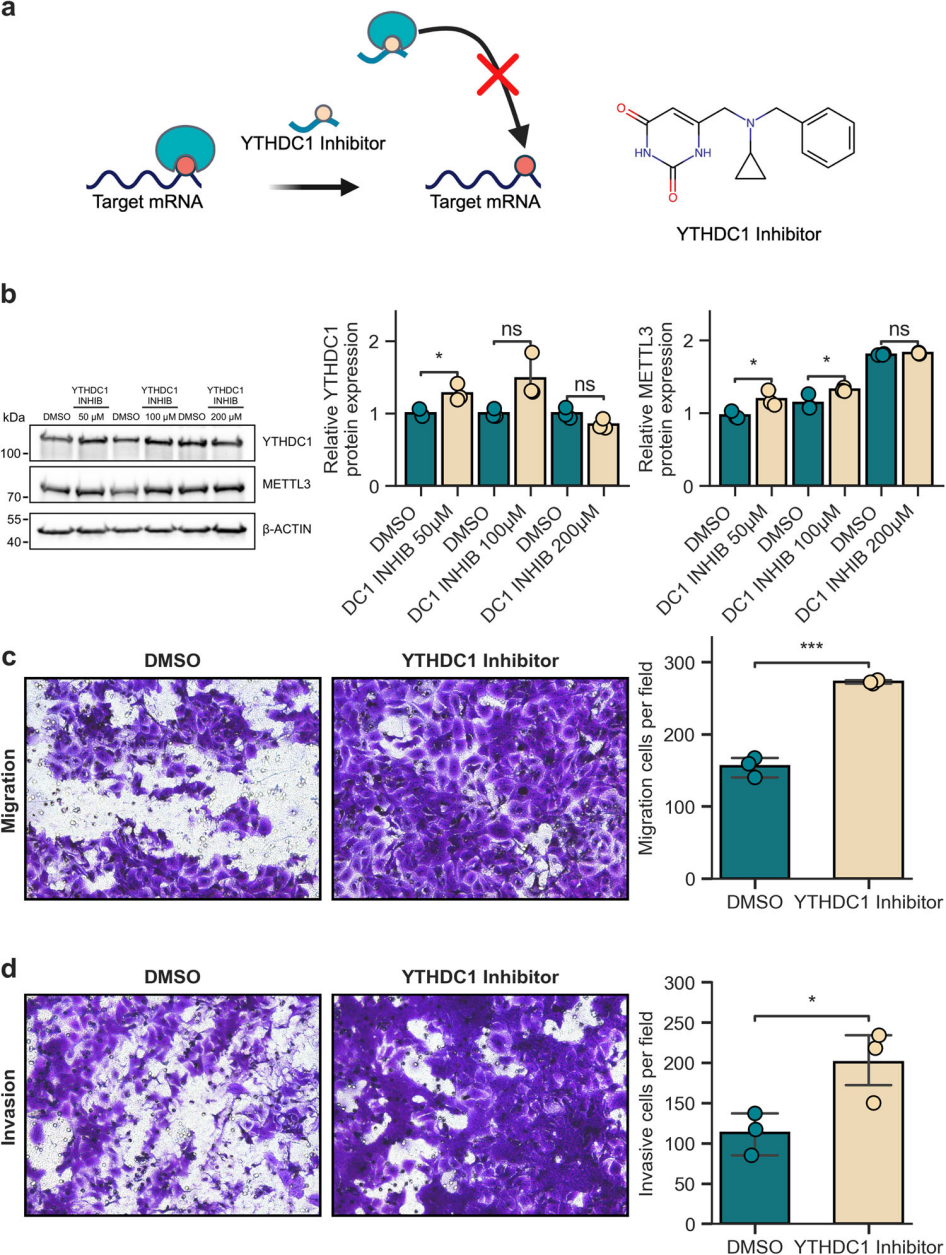
Loss of the m^6^A *reading* function of YTHDC1 enhances urothelial cell migration and invasion. **a** Schematic illustration of our inhibitor approach to disrupt the interaction of YTHDC1 with m^6^A-modified RNAs, with the molecular structure of the YTHDC1 inhibitor depicted on the right. **b** Western blot showing the protein levels of YTHDC1 and METTL3 in wild-type UROtsa cells after treatment with the YTHDC1 inhibitor. The quantification results obtained via ImageJ are shown on the right. *p-value < 0.05; ns: not significant; unpaired two-sided *t*-test. **c** Transwell migration assays of UROtsa cells treated with DMSO (control) or the YTHDC1 inhibitor. Representative images were taken using a 20x objective lens and are displayed on the left, while the quantification and statistics of relative cell migration in three independent replicates are shown on the right. ***p-value < 0.001, unpaired two-sided t test. **d** Transwell invasion assays for UROtsa cells treated with DMSO (control) or the YTHDC1 inhibitor. Representative images were taken using a 20x objective lens and are displayed on the left, while the quantification and statistics of relative cell invasion in three independent replicates are shown on the right. *p-value < 0.05, unpaired two-sided t test.

To determine whether the observed effects are m^6^A dependent, we treated our YTHDC1-depleted cell model with STM2457, a selective catalytic inhibitor of the m^6^A *writer* METTL3^47^. The results revealed a significant (*p*-value < 0.01, two-tailed Student’s *-* test) reduction in the clonogenicity of the YTHDC1-depleted cells (Supplementary Fig. 2g). Finally, to determine whether the metastatic capacity observed in this model upon YTHDC1 downregulation specifically depends on the loss of its m^6^A *reading* function, we also treated wild-type UROtsa cells with a YTHDC1 inhibitor (Fig. 3a), which effectively interferes with the interaction between YTHDC1 and m^6^A^21,48,49^. The compound did not alter the expression of either YTHDC1 or METTL3 (Fig. 3b) but caused a notable increase in the migration and invasion capacities of the cells (Fig. 3c, d). Together, these findings further support the notion that the loss of the YTHDC1 *reader* function promotes the metastatic potential of UCB.

### YTHDC1-depleted urothelial cells promote metastasis at the transcriptomic level

**Fig. 4:**
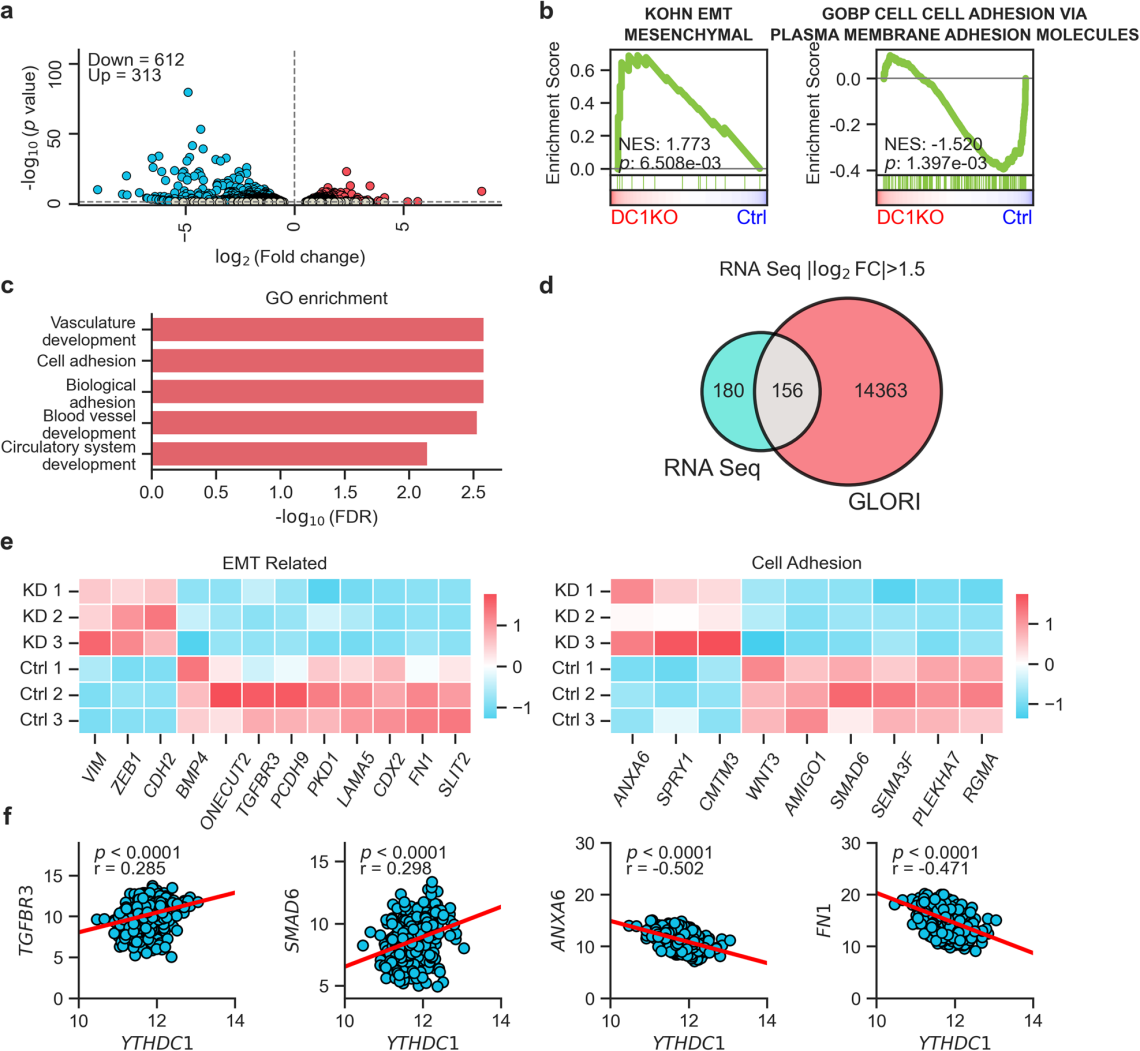
Transcriptomic analysis revealed enrichment of metastasis-related functions in YTHDC1-depleted urothelial cells. **a** Volcano plot illustrating the significantly altered transcripts in YTHDC1-depleted cells compared with Ctrl cells. Downregulated genes are shown in blue, whereas upregulated genes are shown in red. *q* < 0.05. **b** GSEA plots demonstrating the specific dysregulation of gene sets associated with EMT and cell adhesion in YTHDC1-depleted cells compared with control UROtsa cells. The plots display the normalized enriched score (NES) and corresponding *p*-values. **c** GO analysis of the DEGs between YTHDC1-depleted and control UROtsa cells, highlighting the enrichment of metastasis-related processes. **d** Venn diagram showing the overlap between the differentially expressed transcripts ( | Log_2_Foldchange (FC)| > 1.5, *q* < 0.05) upon YTHDC1 depletion in UROtsa cells and those known to be m^6^A modified, as recently determined by GLORI mapping^52^. **e** Heatmaps presenting the expression levels of overlapping transcripts displayed in (**d**), specifically focusing on the terms related to metastasis from the GO analysis. **f** Transcripts in (**e**) exhibiting the highest Pearson correlation with *YTHDC1* expression in TCGA-BLCA samples, with *p*-values and correlation efficiencies (r) shown in the plots.

To better understand the effect of YTHDC1 depletion on the transcriptome, we used RNA-seq analysis of control and YTHDC1-depleted UROtsa cells. In cells with reduced YTHDC1 levels, we detected robust transcriptional changes, identifying 313 significantly (*q* < 0.05) upregulated genes and 612 downregulated genes (Fig. 4a and Supplementary Fig. 3a). Gene set enrichment analysis (GSEA) revealed the specific deregulation of genes associated with EMT, cell adhesion and extracellular matrix (ECM)-related pathways (Fig. 4b and Supplementary Fig. 3b), which is consistent with the notion that lower YTHDC1 levels lead to increased EMT and decreased cell adhesion in UROtsa cells. These results were further supported by gene ontology (GO) analysis, which revealed not only the enrichment of genes associated with cell adhesion but also with vascularization (Fig. 4c), another function that is necessary for tumor metastasis^50,51^. Finally, upon YTHDC1 depletion, we observed significant upregulation of the expression levels of typical p-EMT genes^5^, including *PDPN*, *TNC*, *LAMB3*, *VIM*, and *ANXA5* (Supplementary Fig. 3c).

In further analyses, we investigated the m^6^A methylation status of differentially expressed transcripts. Using a published base-resolution map of m^6^A as a reference^52^, we found that 156 out of the 335 differentially expressed transcripts were methylated (Fig. 4d). Further GO analysis of this subset also revealed enrichment in metastasis-related processes related to cell adhesion (Supplementary Fig. 3d). Furthermore, the expression of mesenchymal markers and EMT activators, including *VIM*, *ZEB1* and *CDH2*, was increased, whereas the expression of EMT suppressors, such as *ONECUT2*, *TGFBR3* and *PCDH9*, was reduced in YTHDC1-depleted cells (Fig. 4e, left). The combined upregulation of positive EMT regulators and downregulation of negative EMT regulators conceivably promotes the EMT process. Similar observations were made for genes related to reduced cell adhesion, such as *ANXA6*, *SPRY1* and *CMTM3*, which presented increased expression levels in YTHDC1-depleted cells, whereas cell adhesion-promoting genes, such as *WNT3*, *AMIGO1* and *SMAD6*, were downregulated (Fig. 4e, right). Importantly, similar effects were also detected in UCB samples from the TCGA dataset (Fig. 4f, and Supplementary Fig. 3e, f). Taken together, the results of our RNA-seq analysis of UROtsa cells with reduced levels of YTHDC1 further confirmed that the loss of function of this m^6^A *reader* led to the deregulation of metastasis-related transcripts.

### Identification of YTHDC1-bound transcripts in urothelial cells

**Fig. 5:**
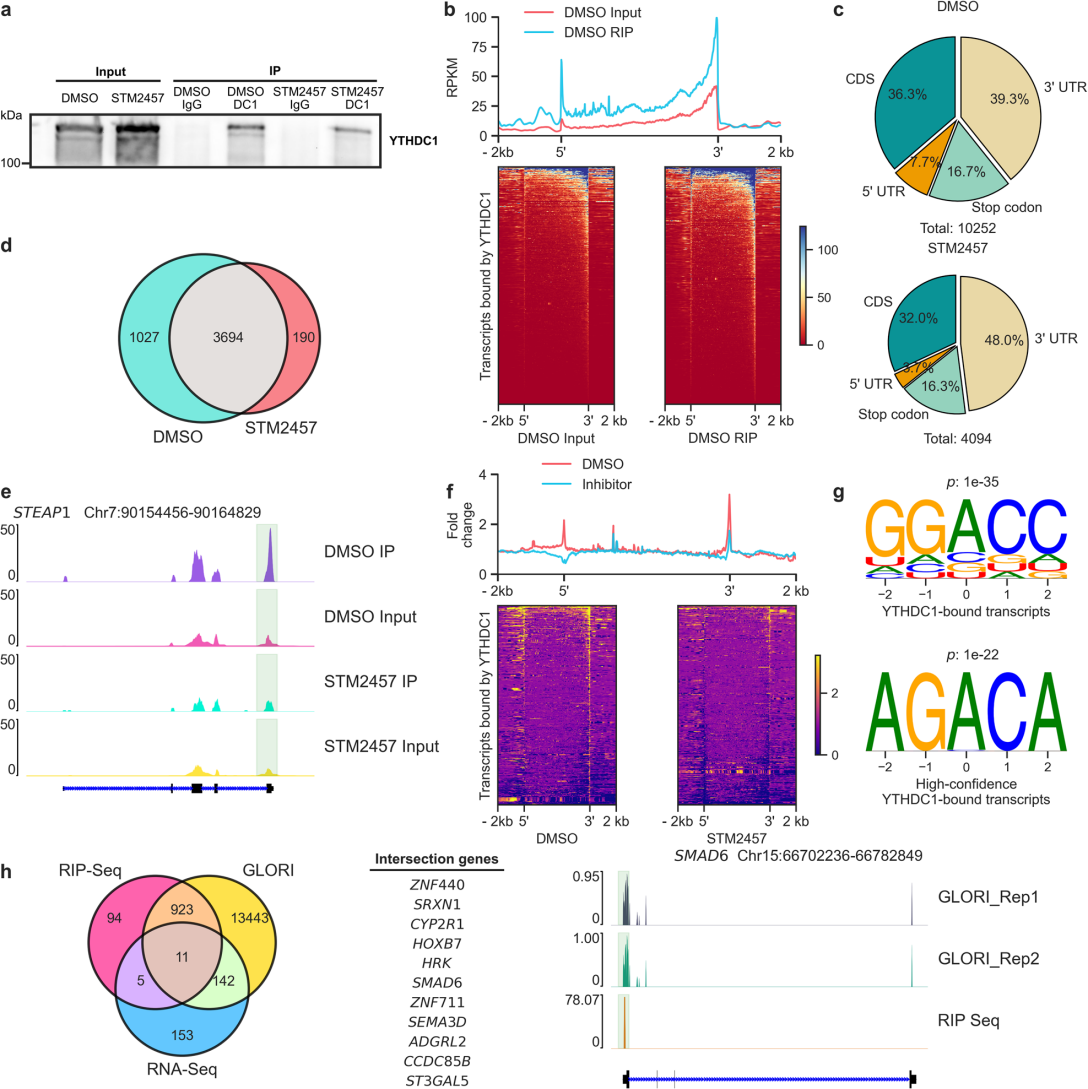
Identification of YTHDC1 binding targets in urothelial cells via RIP-seq. **a** Representative Western blot showing YTHDC1 protein abundance in DMSO- and STM2457-treated wild-type UROtsa cells. IgG was used as an IP control. **b** Read counts (upper panel) from YTHDC1 RIP-seq data in the DMSO group, demonstrating enriched YTHDC1 binding across all transcripts. Blue line: YTHDC1 pulldown; red line: input signal. Heatmap (lower panel) showing enrichment of YTHDC1-bound transcripts in the 5’ to 3’ direction. Each row corresponds to a transcript bound by YTHDC1, where the intensity of the color reflects the enrichment level, with the scale bar shown on the right. **c** Pie charts displaying the distribution of YTHDC1 RIP-seq peaks in the DMSO and STM2457 groups (q < 0.01). **d** Venn diagram indicating the number of YTHDC1 binding targets in the DMSO and STM2457 treatment groups. *p*-value < 0.05. **e** Representative genome tracks depicting an example gene, STEAP1, bound by YTHDC1, with lower peaks in the STM2457 treatment group than in the DMSO group. **f** Heatmaps illustrating enriched YTHDC1 binding (normalized against the input control) in the DMSO and STM2457 treatment groups for all high-confidence YTHDC1-bound transcripts (determined by Diffbind, log_2_fold change < −1, *p*-value < 0.05). **g** Upper row: enriched motifs in YTHDC1-bound sequences identified by RIP-seq in the DMSO group (*p*-value = 1e-35); lower row: enriched motifs in YTHDC1-bound sequences identified by RIP-seq in the high-confidence group (*p*-value = 1e-22). **h** Venn diagram showing the intersection among high-confidence YTHDC1-bound transcripts, m^6^A-modified transcripts, and differentially expressed transcripts in YTHDC1-depleted cells ( | log_2_fold change | > 1.5, *q*-value < 0.05). The intersecting genes are listed in the middle, and representative peaks (signal intensity) are shown in the right panel to demonstrate the overlap between m^6^A peaks (GLORI^52^) and YTHDC1 binding peaks (RIP-seq).

To map the binding of YTHDC1 to mRNAs, we performed RIP-seq in wild-type UROtsa cells (Fig. 5a). As a control, we treated UROtsa cells with the METTL3 inhibitor STM2457^47^, which removes a substantial portion of the m^6^A marks on mRNAs and should thus reduce the number of mRNAs bound by YTHDC1 (Fig. 5b and Supplementary Fig. 4a). This approach identified 10,252 annotated YTHDC1 RIP-seq peaks in wild-type UROtsa cells, which decreased to 4,094 after treatment with STM2457 (Fig. 5c and Supplementary Fig. 4b). Consistent with the canonical distribution of m^6^A modifications^52,53^, the detected YTHDC1-binding peaks presented high levels of enrichment in the CDS, stop codon and 3’ untranslated region (3’ UTR) (Fig. 5c). Subsequent identification of differentially bound transcripts between control (DMSO-treated) and STM2457-treated cells allowed us to define 1,027 high-confidence YTHDC1-binding mRNA targets that were lost following treatment with the METTL3 inhibitor (Fig. 5d, e and Supplementary Fig. 4c). Interestingly, the corresponding high-confidence peaks were located not only at the 3’UTR but also at the 5’UTR (Fig. 5f), which presented comparably low methylation frequencies^52^. Moreover, YTHDC1 mRNA-binding sites were enriched in GGACC motifs corresponding to the canonical m^6^A “DRACH” motif (D = A/G/U, R = A/G and H = A/C/U)^52^, and the AGACA consensus was enriched in the high-confidence subset (Fig. 5g). This finding is consistent with published findings obtained via different methods^20,54^ and corroborates that YTHDC1 prefers the G(m^6^A)C motif, which distinguishes it from other YTH domain proteins^55^.

Additional analysis revealed that the vast majority of the high-confidence YTHDC1-binding mRNA targets (927 out of 1027) were m^6^A modified in a published reference dataset^52^ (Supplementary Fig. 4d). Notably, a significant fraction of these transcripts were downregulated upon YTHDC1 depletion (Supplementary Fig. 4e, f), suggesting a predominant role for YTHDC1 in mRNA stabilization, which is consistent with observations in other cancer types^21,56^. Gene enrichment analysis of the m^6^A-modified YTHDC1-bound transcripts revealed their associations with processes and pathways such as TGF-β signaling, Hedgehog signaling, ErbB signaling, and Wnt/β-catenin signaling (Supplementary Fig. 4g), which are involved in UCB development and closely related to the EMT process^57–59^.

To delineate a core set of transcripts that are likely regulated by YTHDC1, we next integrated the results obtained from RIP-seq and RNA-seq with the published m^6^A reference dataset^52^, which identified 11 genes (Fig. 5h). Importantly, while most of these genes are related to the etiology of various tumor types^60–62^, some of them, such as *SMAD6*, have also been connected with metastasis^62^. Moreover, the YTHDC1 binding sites of these transcripts consistently overlapped with the positions of m^6^A modifications (Fig. 5h and Supplementary Fig. 5). Taken together, the results of our RIP-seq experiments further support the role of this m^6^A *reader* in driving UCB metastasis and identify a subset of YTHDC1-regulated genes that might play a central role in this process.

### Epitranscriptomic deregulation of *SMAD6* drives invasiveness in UCB

**Fig. 6:**
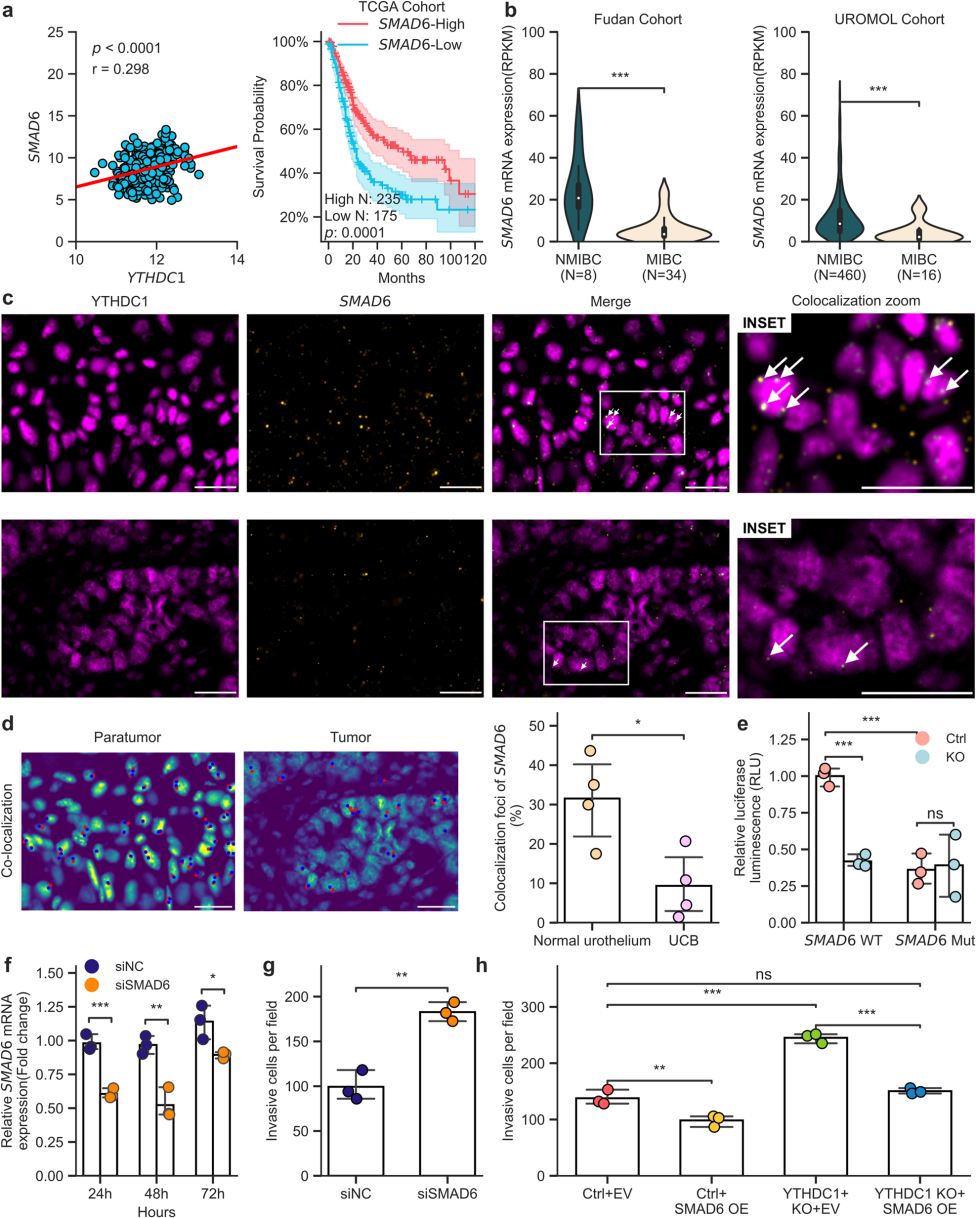
Dysregulation of YTHDC1-*SMAD6* promotes invasion in UCB. **a** Left panel: Pearson correlation analysis between *YTHDC1* expression (Log_2_(normalized counts +1)) and *SMAD6* (Log_2_(normalized counts +1)) in the TCGA-BLCA dataset, with *p* values and correlation coefficients (r) provided. Right panel: Ten-year overall survival analysis of *SMAD6* in the TCGA-BLCA dataset. Log-rank test, *p*-value = 0.0001. **b** Comparison of *SMAD6* expression in NMIBC and MIBC using data from the Fudan and UROMOL cohorts. ***p-value < 0.001, Mann‒Whitney U test. **c** Representative images of *SMAD6* mRNA (yellow) and YTHDC1 protein (magenta) detected in paratumoral (upper row) and tumoral (lower row) FFPE tissues, respectively. The white arrows indicate *SMAD6*-YTHDC1 colocalization. Scale bar = 10 μM. **d** Left panel: Colocalization detection results using Big-FISH of the abovementioned paratumoral and tumoral tissues. Scale bar = 10 μM. Right panel: Quantification of the percentage of colocalized *SMAD6* foci. *p-value < 0.05, paired Student’s *t* test. **e** Relative luciferase activity in control and YTHDC1-depleted UROtsa cells transfected with wild-type or m^6^A-mutant *SMAD6* 5’UTR constructs. Three independent experiments were performed. ***p-value < 0.001, ns: not significant. **f** Time course qPCR analysis of *SMAD6* mRNA expression in UROtsa cells following transfection with nontargeting control siRNA (siNC) or *SMAD6*-targeting siRNA (siSMAD6). The experiments were performed with 3 biological replicates. ***p-value < 0.001, **p-value < 0.01, *p-value < 0.05. **g** Quantification of Transwell invasion assays of UROtsa cells transfected with siNC and siSMAD6 after 24 h. Three biological replicates, **p-value < 0.01. **h** Quantification of Transwell invasion assays of UROtsa Ctrl and YTHDC1-depleted cells transduced with empty vector (EV) and SMAD6 overexpression (OE). ***p-value < 0.001, **p-value < 0.01, ns: not significant. One-way analysis of variance (ANOVA) followed by Tukey’s post hoc test was used.

Finally, we further investigated the role of *SMAD6*, a transcript found to be regulated by YTHDC1 in our cell models (Fig. 5h). Our analysis of the TCGA-BLCA dataset revealed a positive correlation between *SMAD6* and *YTHDC1* expression (Fig. 6a, left), which is consistent with *SMAD6* being a downstream effector of YTHDC1 in UCB. Importantly, we also found that lower *SMAD6* expression levels predict a worse prognosis in UCB patients (Fig. 6a, right) and that *SMAD6* is expressed at lower levels in MIBC patients than in NMIBC patients (Fig. 6b). Our results are therefore consistent with the notion that *SMAD6* downregulation, due to the loss of YTHDC1 *reader* function, promotes invasiveness in UCB.

To verify the direct binding of YTHDC1 to *SMAD6* transcripts, we performed RIP-qPCR in UROtsa cells with or without YTHDC1 inhibitor treatment. The RIP-qPCR results validated the binding between YTHDC1 and *SMAD6* and demonstrated that the binding was disrupted by the YTHDC1 inhibitor (Supplementary Fig. 6a). In further experiments, we identified tumor areas in UCB tissue sections via H&E staining (Supplementary Fig. 6b) and then used simultaneous RNA fluorescence in situ hybridization (FISH) and immunofluorescence (IF) to detect SMAD6 mRNA and YTHDC1 protein, respectively. The results revealed *SMAD6* mRNA foci and YTHDC1 puncta in the cell nucleus, with stronger signals in the peritumoral areas than in the tumoral regions (Fig. 6c and Supplementary Fig. 6c), which is consistent with our previous data. In addition, colocalization between YTHDC1 and *SMAD6* mRNA was also observed and was considerably less pronounced in tumoral tissue (Fig. 6d). To assess whether direct binding to m^6^A is required for YTHDC1-mediated gene regulation, we next transfected our UROtsa models with a luciferase reporter gene fused with the 5’UTR of *SMAD6*, which includes its regulatory m^6^A sites (Supplementary Fig. 6d). Importantly, YTHDC1 depletion significantly (*p*-value < 0.001, two-tailed Student’s *t* test) reduced luciferase activity, but this effect largely disappeared when the m^6^A sites were mutated (Fig. 6e). qPCR analysis also revealed decreased *SMAD6* expression after the cells were treated with the METTL3 inhibitor STM2457 (Supplementary Fig. 6e). Finally, to test the potential direct effect of *SMAD6* downregulation on cell migration and invasion, we silenced *SMAD6* in UROtsa cells through siRNA transfection (Fig. 6f). Our results revealed an increase in key metastasis-related phenotypes (Fig. 6g and Supplementary Fig. 6f, g).

To confirm that these phenotypes are mediated by the YTHDC1-*SMAD6* axis, we performed a variety of functional assays. Our results revealed that *SMAD6* overexpression (Supplementary Fig. 7a) counteracted the increased viability and growth and decreased apoptosis caused by YTHDC1 deficiency in UROtsa cells (Supplementary Fig. 7c–e). Importantly, the increased migration and invasion caused by YTHDC1 deficiency were also reversed by *SMAD6* overexpression (Fig. 6h and Supplementary Fig. 7f–g). Moreover, in two additional bladder cancer cell lines, UM-UC-3 and RT112, *SMAD6* knockdown (Supplementary Fig. 7b) partially reversed the reduction in cell viability (Supplementary Fig. 7c) and partially reversed the increase in caspase3/7 activity caused by *YTHDC1* overexpression (Supplementary Fig. 7e, right). Similarly, the decreased migration and invasion caused by *YTHDC1* overexpression could be partially restored by *SMAD6* knockdown (Supplementary Fig. 7h). It is reasonable to assume that the observed phenotype reversal was limited by the pronounced *YTHDC1* overexpression in our inducible system. Taken together, these data demonstrate that YTHDC1 regulates *SMAD6* expression in UCB in a m^6^A-dependent manner and thus reveal a mechanism through which downregulation of an epitranscriptomic *reader* can promote tumor invasiveness.

## Discussion

This study describes a novel m^6^A-dependent YTHDC1–*SMAD6* axis that acts as a critical regulator of cell invasiveness, highlighting the importance of epitranscriptomic mechanisms in UCB. We demonstrated that YTHDC1 was downregulated in more invasive MIBC and inversely correlated with EMT genes. The functional inhibition of YTHDC1 promoted migration and invasion in urothelial cell models. Through the integration of RNA-seq, RIP-seq and published m^6^A datasets, we identified a subset of direct targets of YTHDC1, including *SMAD6*. Notably, *SMAD6* transcripts exhibited reduced colocalization with YTHDC1 in UCB tissues compared with adjacent normal tissues, indicating that the YTHDC1–*SMAD6* interaction was disrupted during cancer progression. Our findings establish *SMAD6* as a critical m^6^A-regulated target of YTHDC1 and suggest that its downregulation contributes to UCB invasiveness. These findings provide novel insights into the epitranscriptomic metastasis network in UCB.

Our analysis of multiple patient cohorts and IHC experiments revealed lower YTHDC1 levels in MIBC patients. Approximately 25% of bladder cancers are diagnosed at the MIBC stage^63^, which is associated with aggressive behavior, poor prognosis, and limited response to systemic therapy^2^. Our results thus expand recent findings that linked the downregulation of *YTHDC1* with poor prognosis in UCB patients in the TCGA dataset^23^. The fact that no differences were detected between the Ta and Tis stages of NMIBC via IHC assays suggests that the loss of YTHDC1 during the transition from NMIBC to MIBC may facilitate the invasion of bladder cancer cells into the muscle layer.

Our observations in multiple cell lines and human tissue samples support a tumor-suppressing role for YTHDC1 in bladder cancer, which is consistent with previous findings demonstrating enhanced tumor growth and chemoresistance in YTHDC1-depleted xenografts^23,24^. Furthermore, the decreased expression of *YTHDC1* is correlated with the expression of markers of EMT, a critical process that promotes cancer invasion and metastasis^64^. In agreement with recent reports showing that p-EMT phenotypes are more prevalent in MIBC than in NMIBC^65^, we found lower *YTHDC1* expression in tumors with a high p-EMT state. Importantly, YTHDC1 depletion in UROtsa cells upregulated the expression of the main p-EMT markers^5^ and enhanced migration and invasion. Collectively, these findings suggest that YTHDC1 downregulation promotes tumor invasiveness in UCB by facilitating p-EMT.

We also aimed to explore the ability of YTHDC1 to modulate cancer invasiveness through m^6^A-dependent mechanisms. To this end, we used a recently developed chemical inhibitor that selectively disrupts the interaction of YTHDC1 with m^6^A-modified transcripts^13,49^. This inhibitor has a dissociation constant of 50 μM, and its binding to other m^6^A *readers*, such as YTHDF1-3 and YTHDC2, is negligible^66^. Importantly, treatment of urothelial cells with this YTHDC1 inhibitor recapitulated the increased migration and invasion capabilities observed after YTHDC1 depletion without altering the protein levels of YTHDC1 or the m^6^A *writer* METTL3. These findings indicate that a correct YTHDC1 m^6^A *reading* capacity is essential for preventing the development of the metastatic potential of urothelial cells.

Moreover, we performed RIP-seq in the presence and absence of the METTL3 inhibitor STM2457^47^. Interestingly, while YTHDC1 RIP-seq peaks were enriched primarily in CDSs, stop codons and 3’UTRs, which aligns with canonical m^6^A patterns, high-confidence YTHDC1-binding peaks also localized to 5’UTR regions. The role of the 5’UTR in translation initiation remains under debate. Recent research has suggested that single 5’UTR m^6^A modifications near start codons have a minimal impact on translation yields or the kinetics of initiation complex assembly^67^. Nevertheless, YTHDC1 binding to 5´UTR regions may influence other protein interactions and modulate translation efficiency in a context-dependent manner.

Furthermore, we demonstrated a reduced interaction between YTHDC1 and *SMAD6* mRNA in UCB via RNA FISH and IF codetection in patient samples, suggesting that loss of the YTHDC1 m^6^A *reader* function may contribute to UCB pathogenesis. This codetection approach provides direct visualization of protein‒RNA interactions and represents a robust approach for future epitranscriptomic investigations to delineate the impact of m^6^A *readers* in various biological settings. By integrating our RNA-seq and RIP-seq experiments with a published m^6^A dataset^52^, we identified and validated *SMAD6* mRNA as a YTHDC1 target that is potentially involved in UCB metastasis.

SMAD6 is an inhibitory SMAD family member that negatively regulates TGF-β signaling pathways by competing with receptor-regulated SMADs^68^. As a key regulator of the TGF-β pathway, it has been shown to inhibit EMT in various cancers^62,69^ and was recently shown to suppress EMT and metastasis in bladder cancer^70^. Consistently, our study revealed that the loss of YTHDC1 led to reduced levels of *SMAD6*, thereby promoting EMT and metastasis in UCB. The YTHDC1–*SMAD6* axis was further supported by rescue experiments, where SMAD6 overexpression counteracted the prometastatic effects of YTHDC1 depletion.

In summary, this study provides an unbiased view of YTHDC1 and highlights its function as a m^6^A *reader* in UCB invasiveness. We revealed the role of YTHDC1 in modulating the invasiveness of UCB and identified *SMAD6* as a key downstream target. These findings provide a foundation for future studies aiming to further characterize the YTHDC1–*SMAD6* axis as a potential therapeutic target to prevent the transition of NMIBC to MIBC. Additionally, the observation of fewer YTHDC1 puncta in UCB suggests the possibility of exploring the formation and function of YTHDC1 nuclear condensates in UCB, particularly regarding their influence on mRNA stability and EMT-related processes. Overall, our work contributes to the understanding of the role of m^6^A-binding proteins in cancer formation and progression.

## Supplementary information

Supplementary Information

## Data Availability

The data generated in this study have been deposited in the GEO database under the accession numbers GSE266223 and GSE266224.
