# Supplementary material for: Loss of YTHDC1 m6A reading function promotes invasiveness in urothelial carcinoma of the bladder
Source: Exp Mol Med. 2025 Jan 1;57(1):118–30. doi: 10.1038/s12276-024-01377-x (PMC11799412; doi:10.1038/s12276-024-01377-x)
Supplement: Supplementary file 1 — Supplementary Information [file 12276_2024_1377_MOESM1_ESM.pdf]

Title: Loss of YTHDC1 m^{6}A *reading* function promotes invasiveness in urothelial carcinoma of the bladder

Author List: Jinyun Xu[1], Jonas Koch[1], Claudia Schmidt[2], Malin Nientiedt[3], Manuel Neuberger[3], Philipp Erben[3], Maurice Stephan Michel[3], Manuel Rodríguez-Paredes[1] & Frank Lyko[1]

Authors' Affiliations:

[1]Division of Epigenetics, DKFZ-ZMBH Alliance, German Cancer Research Center, 69120 Heidelberg, Germany;

[2]Core Facility Unit Light Microscopy, German Cancer Research Center, 69120 Heidelberg, Germany;

[3]Department of Urology and Urosurgery, Medical Faculty Mannheim, University of Heidelberg, 68167 Mannheim, Germany

Corresponding author:

Frank Lyko, Division of Epigenetics, DKFZ-ZMBH Alliance, German Cancer Research Center, 69120 Heidelberg, Germany. Tel: +49 6221423800, E-mail: f.lyko@dkfz.de

Table of Contents

Supplementary Figures

Supplementary Fig. 1 YTHDC1 expression analysis in UCB and its correlation with EMT markers.

Supplementary Fig. 2 Functional characterization of YTHDC1 in urothelial and UCB cell lines.

Supplementary Fig. 3 Transcriptomic analysis of YTHDC1-depleted UROtsa cells and correlation with EMT processes.

Supplementary Fig. 4 RIP-seq analysis of YTHDC1-bound transcripts.

Supplementary Fig. 5 Visualization of m^6A peaks and YTHDC1 binding sites across intersected genes.

Supplementary Fig. 6 Epitranscriptomic modulation of *SMAD6* enhances tumor invasion in UCB.

Supplementary Fig. 7 SMAD6 overexpression reverses the phenotype of YTHDC1 depletion in UROtsa cells.

Supplementary Tables

Supplementary Table 1 The clinicopathological characteristics of patients across different cohorts.

Supplementary Table 2 Overview of the FFPE samples used for IHC and RNA FISH experiments.

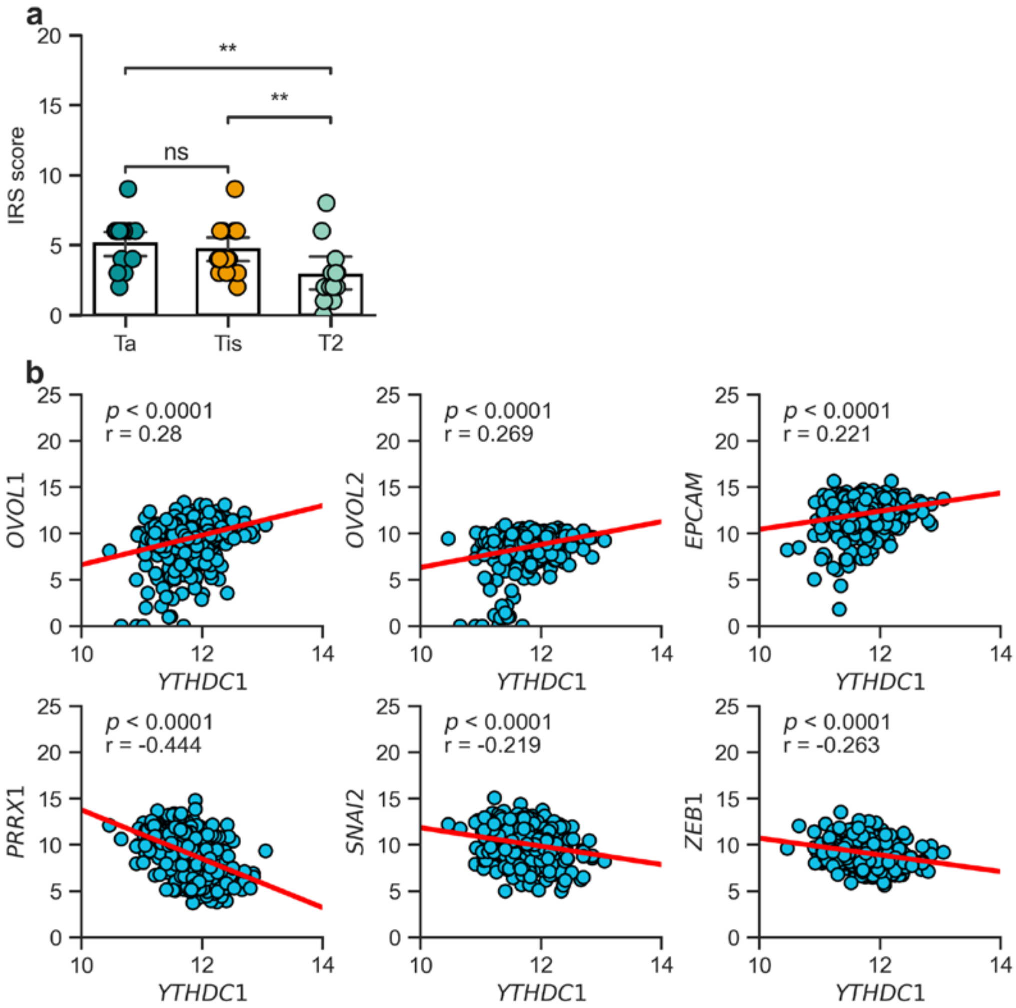

Supplementary Fig. 1. YTHDC1 expression analysis in UCB and its correlation with EMT markers. (a) Quantitative analysis of YTHDC1 IHC assays, using the IRS method comparing different stages of UCB. **: p value<0.01, ns: not significant. Mann-Whitney-U test. **(b)** Correlation analysis between *YTHDC1* expression (Log_2(normalized counts +1)) and canonical EMT markers (Log_2(normalized counts +1)) in the TCGA-BLCA dataset, with p values and correlation coefficients (r) provided.

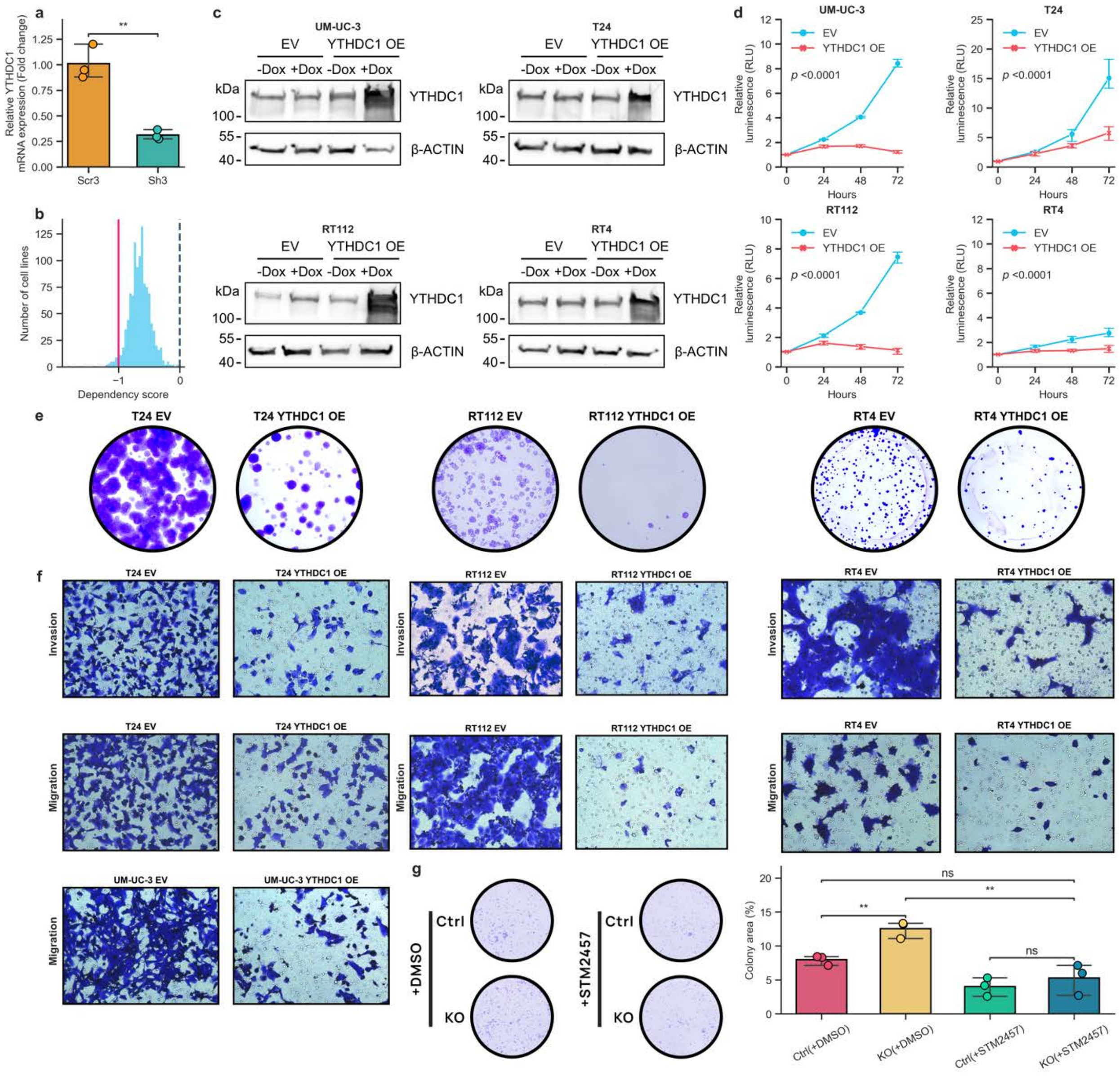

Supplementary Fig. 2. Functional characterization of YTHDC1 in urothelial and UCB cell lines. (a) qPCR analysis of *YTHDC1* level upon using shRNAs in UROtsa cells. **: *p* value < 0.01, unpaired two-sided t test. **(b)** DepMap data analysis of *YTHDC1* dependencies in all cell lines. The x-axis indicates the gene dependency scores. The lower this score, the higher the probability that a gene is essential. A cell line is considered pan-essential if it has a dependency score close to -1. **(c)** Western blot of YTHDC1 after transduced with empty vector (EV) or YTHDC1 overexpression (OE) vector in UM-UC-3, T24, RT112 and RT4 cells, respectively. **(d)**

Cell viability in UM-UC-3, T24, RT112, RT4 cells after overexpressing YTHDC1, as analyzed by the cell-titer-glo assay. p value < 0.0001, two-way analysis of variance (ANOVA). Experiments were performed in biological and technical triplicates. **(e)** Representative pictures of the Colony formation assays in T24, RT112, RT4 EV and YTHDC1 OE cells, respectively. **(f)** Representative pictures of the Transwell assays for multiple BLCA EV and YTHDC1 OE cells. First and second row: Transwell invasion and migration assays of T24, RT112 and RT4 EV and YTHDC1 OE cells, respectively. Third row: representative pictures of Transwell migration assays in UM-UC-3 EV and YTHDC1 OE cells. **(g)** Representative images of colony formation assays contrasting control UROtsa YTHDC1 cells with YTHDC1-depleted cells, both with and without STM2457 treatment (left panel). Quantification was performed using the ColonyArea plug-in in ImageJ (right panel). **: p value < 0.01, ns: not significant, unpaired two-sided t test.

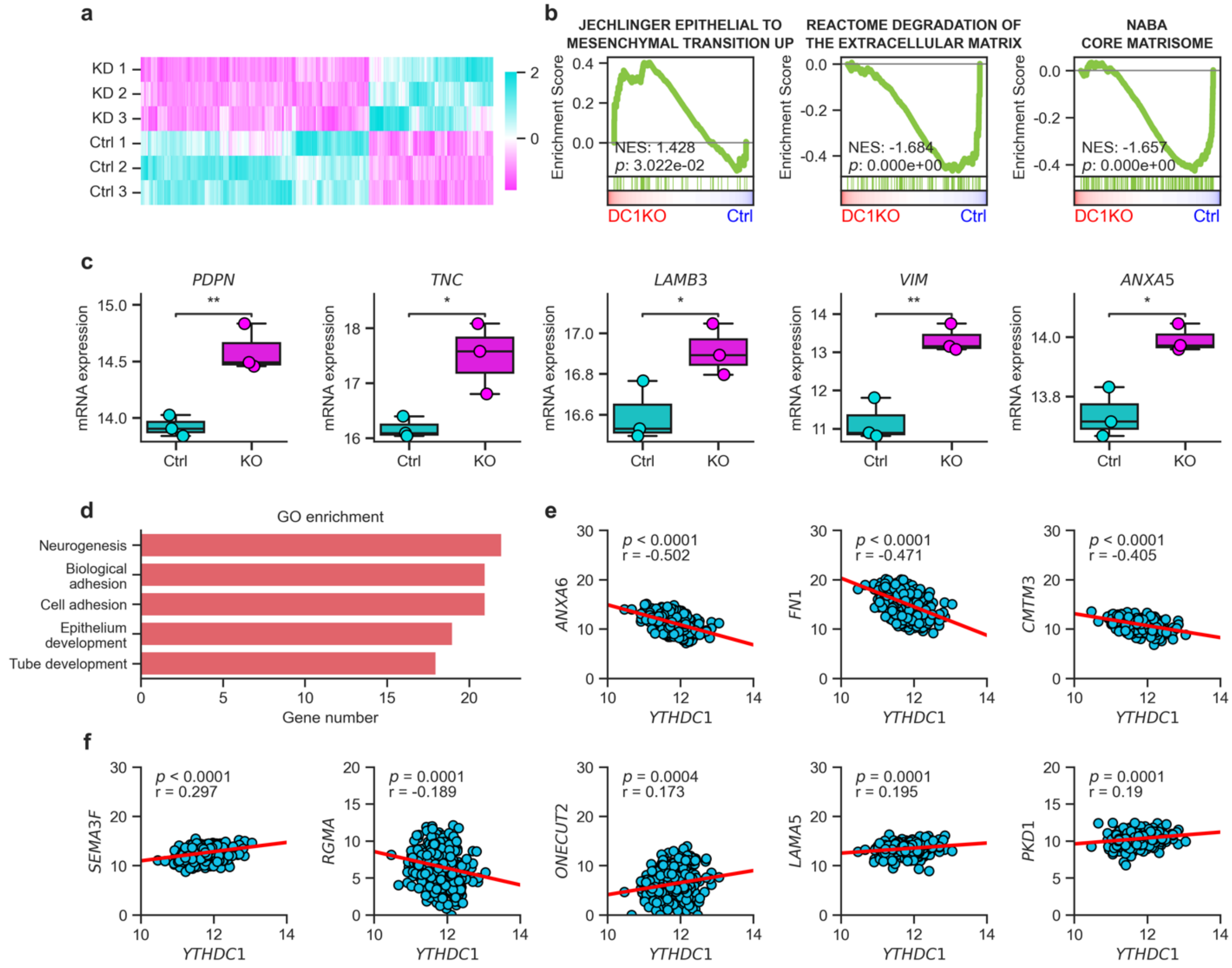

Supplementary Fig. 3. Transcriptomic analysis of YTHDC1-depleted UROtsa cells and correlation with EMT processes. (a) Heatmap displaying the significantly changed transcripts in UROtsa YTHDC1-depleted cells compared to UROtsa Ctrl cells. Down-regulated genes are shown in blue, while up-regulated genes are shown in magenta. $q < 0.05$. **(b)** GSEA plots demonstrating the specific dysregulation of gene sets associated with EMT, extracellular matrix and core matrisome in YTHDC1-depleted cells, compared to UROtsa Ctrl. cells. The plots display the normalized enriched score (NES) and corresponding *p* values. **(c)** The relative mRNA expression (Log_2(normalized counts +1)) of main p-EMT genes in UROtsa Ctrl. and YTHDC1-depleted cells. **: *p* value < 0.01, *: *p* value < 0.05. **(d)** GO analysis of the intersected genes of differentially expressed transcripts after YTHDC1 depletion in UROtsa cells and m^6A-

mapping (GLORI) dataset, highlighting enrichment of cell adhesion processes. **(e)-(f)** Transcripts in Fig. 4e exhibiting the highest positive correlation with *YTHDC1* expression (Log_2(normalized counts +1)) in TCGA-BLCA. Pearson correlation, with *p* value and correlation efficient (r) provided in the plots.

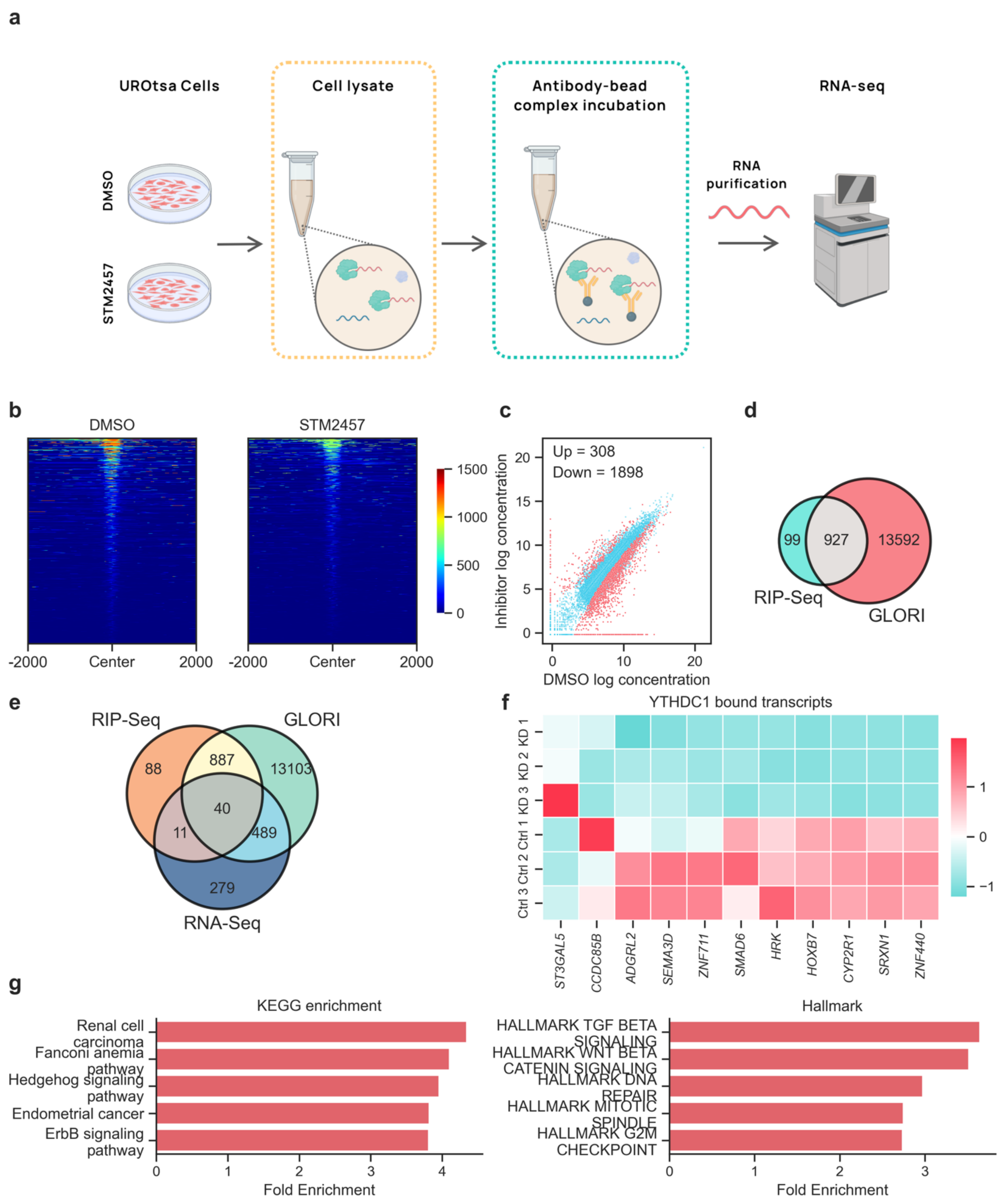

Supplementary Fig. 4. RIP-seq analysis of YTHDC1-bound transcripts. (a) Schematic representation of the RIP-seq experimental design and workflow. The diagram illustrates the main steps involved in the RIP seq and the experimental conditions. **(b)** Heatmaps illustrating

RIP-seq peak enrichment across all YTHDC1-bound transcripts in both DMSO and STM2457-treated groups. Each row corresponds to a unique YTHDC1 peak, with the color intensity indicative of the level of enrichment. **(c)** MA plot of differentially bound sites identified by Diffbind with an FDR threshold of <0.05. Each point on the plot represents a unique binding site, with points in red highlighting those sites determined by Diffbind to be statistically significantly differentially bound (*q* value < 0.05). **(d)** Venn diagram illustrating the overlap between high-confidence YTHDC1-bound transcripts (Log_2foldchange < -1, *p* value < 0.05) and m^6A-modified transcripts as identified in the m^6A mapping dataset. **(e)** Venn diagram showing the intersection of m^6A-modified YTHDC1-bound transcripts that significantly change (*q* < 0.05) following YTHDC1 depletion. **(f)** Heatmap demonstrating changes in m^6A-modified YTHDC1-bound transcripts after YTHDC1 depletion. **(g)** KEGG (left panel) and HALLMARK (right panel) pathway enrichment of the intersected transcripts identified in (d).

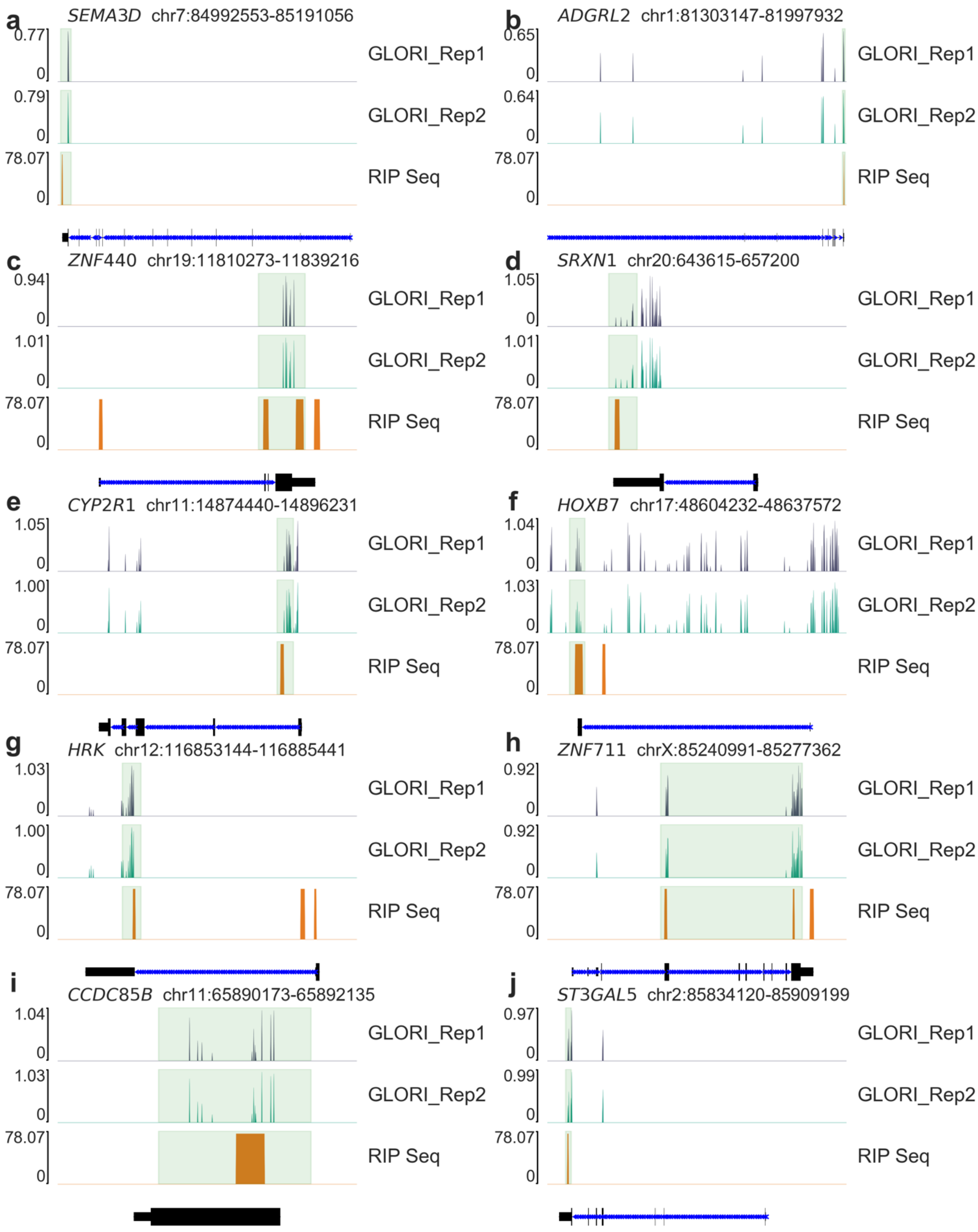

Supplementary Fig. 5. Visualization of m^{6}A peaks and YTHDC1 binding sites across intersected genes. (a)-(j) Genome tracks showing representative peaks for the all genes

identified in the intersection of RNA-seq, YTHDC1 RIP-seq and the published GLORI dataset. For each gene, m^6A peaks identified by GLORI (purple and green) and YTHDC1 binding sites determined by RIP-seq (yellow) are displayed, and the overlapped region are indicated by the light green region. Gene name and genomic regions are showed above each panel.

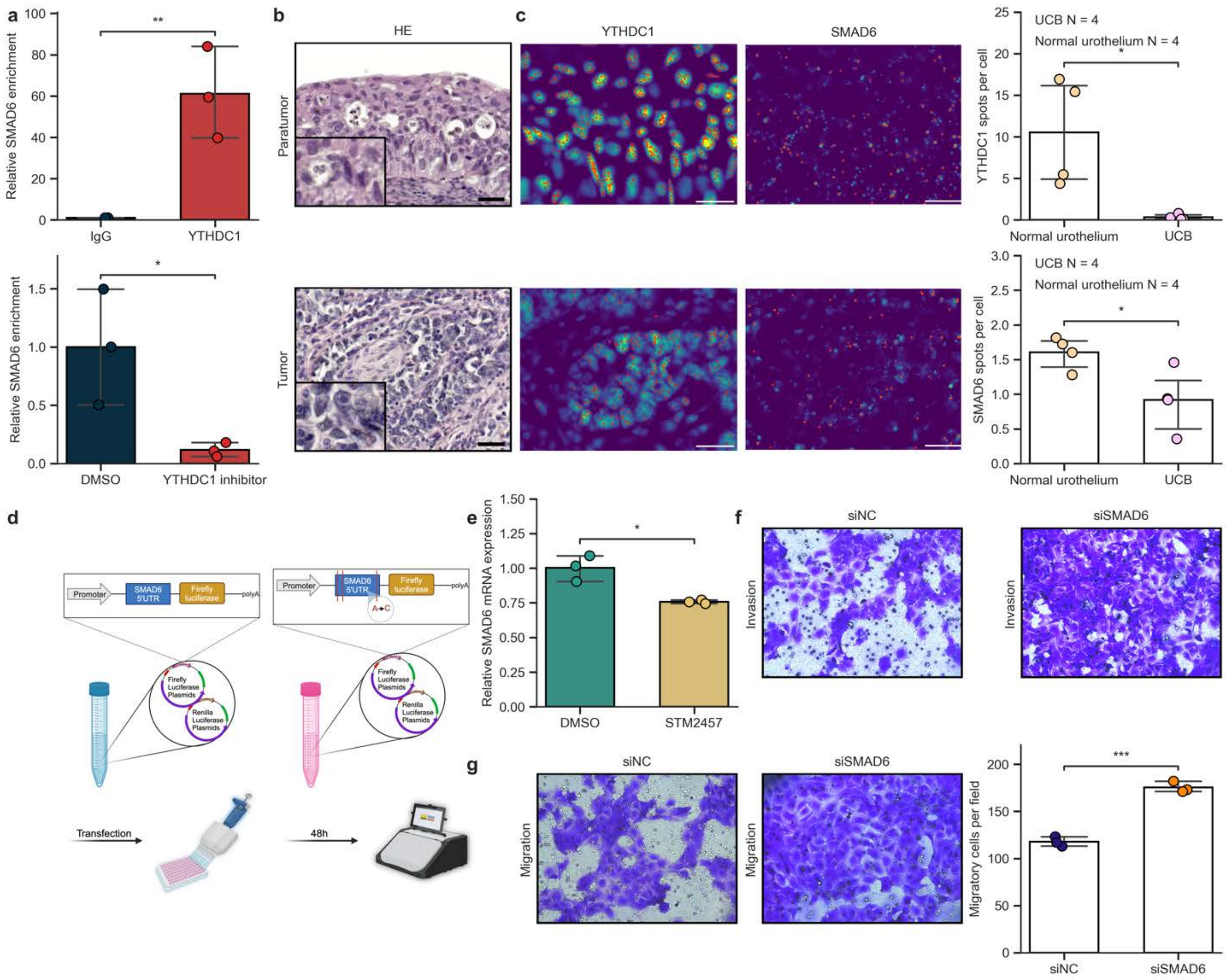

Supplementary Fig. 6. Epitranscriptomic modulation of *SMAD6* enhances tumor invasion in UCB. (a) Validation of the bindings between YTHDC1 and *SMAD6* in UROtsa cells using YTHDC1 specific antibody by RIP-qPCR. **(b)** Representative H&E staining images of the paratumor (upper panel) and tumor (lower panel) FFPE tissues. Scale bar = 20 μM. **(c)** Left panel: Detection of YTHDC1 and *SMAD6* using Big-FISH in paratumor (upper panel) and tumor (lower panel) tissues, with detected spots indicated by red circles. Scale bar = 100 μM. Right panel: Quantification of YTHDC1 and *SMAD6* spots per cell. *: p value < 0.05, paired Student's t test. **(d)** The scheme of the Luciferase assays performed. **(e)** qPCR analysis of *SMAD6* mRNA expression upon STM2457 treatment in UROtsa cells. *: p value < 0.05. **(f)** Representative images of Transwell invasion assays for UROtsa cells transfected with non-targeting control

siRNA (siNC) and *SMAD6*-targeting siRNA (siSMAD6) after 24 hours. **(g)** Transwell migration assays of UROtsa cells transfected with siNC and siSMAD6 after 24 hours (left panel) with quantification of invasive cells per field (right panel). 3 biological replicates were used in this experiment. ***: p value < 0.001. The images were taken under 20x objective lens.

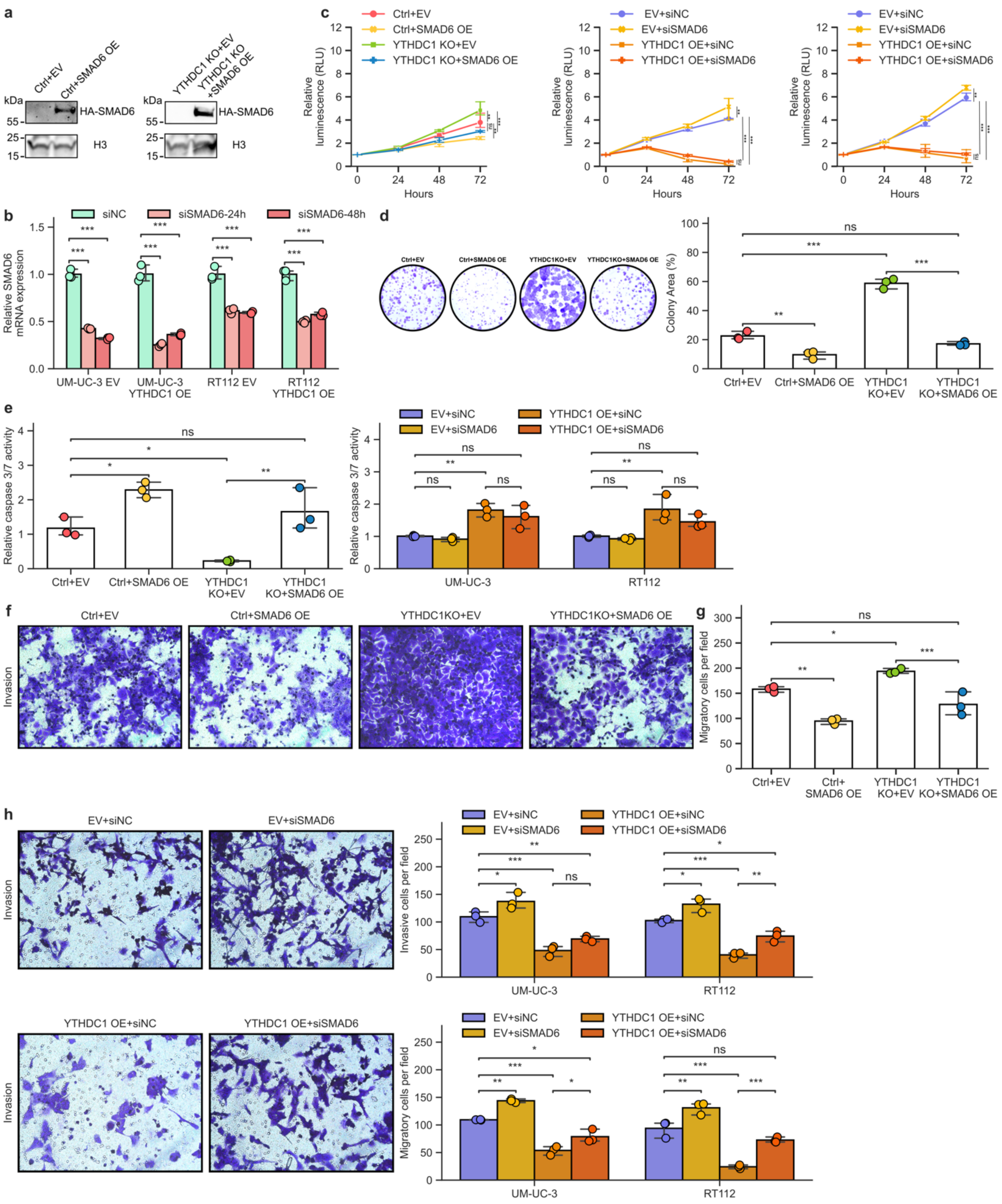

Supplementary Fig. 7. SMAD6 overexpression reverses the phenotype of YTHDC1 depletion in UROtsa cells.

(a) Western blot analysis of SMAD6 overexpression in both UROtsa control (Ctrl) and YTHDC1 depleted (YTHDC1 KO) cells. **(b)** Time course qPCR analysis of *SMAD6* mRNA expression in UM-UC-3 and RT112 cells with empty vector (EV) or YTHDC1 overexpression (OE) following transfection with non-targeting control siRNA (siNC) and *SMAD6*-targeting siRNA (siSMAD6). The experiments were performed in 3 biological replicates. ***: *p* value < 0.001. **(c)** Cell viability in UROtsa YTHDC1 depleted cells with SMAD6 overexpression (left panel) and in UM-UC-3/RT112 YTHDC1 overexpressed cells upon SMAD6 knock-down (right panel), assessed by the cell-titer-glo assay. ***: *p* value < 0.001, **: *p* value <0.01, ns: not significant, two-way analysis of variance (ANOVA). **(d)** Colony formation assays in UROtsa YTHDC1 Ctrl. and YTHDC1 KO cells with EV. or SMAD6 OE. Representative images (left) and quantification measure by ColonyArea (right) are shown. n=3, ***: *p* value <0.001, **: *p* value <0.01, one-way analysis of variance (ANOVA) followed by Tukey's post hoc test. **(e)** Apoptosis levels in UROtsa YTHDC1 Ctrl and KO cells with EV or SMAD6 OE (left) and in UM-UC-3/RT112 EV and YTHDC1 OE cells with siNC or siSMAD6 (right), measured by Caspase-3/7-Glo assays. n=3, **: *p* value <0.01, *: *p* value < 0.05, ns: not significant, one-way ANOVA followed by Tukey's post hoc test. **(f)** Representative pictures of Transwell invasion assays for YTHDC1 Ctrl. and YTHDC1 KO cells with EV or SMAD6 OE. **(g)** Quantification and statistical analysis of Transwell migration assays for UROtsa YTHDC1 Ctrl. and YTHDC1 KO cells with EV or SMAD6 OE. ***: *p* value <0.001, **: *p* value < 0.01, *: *p* value <0.05, ns: not significant. one-way ANOVA followed by Tukey's post hoc test. **(h)** Representative images of Transwell invasion assays in UM-UC-3 EV and YTHDC1 OE cells with siNC or siSMAD6 (left panel, 20x objective). Quantification of Transwell migration and invasion assays in UM-UC-3/RT112 EV and YTHDC1 OE cells with siNC or siSMAD6 (right panel). n=3, ***: *p* value <0.001, **: *p* value <0.01, *: *p* value <0.05, ns: not significant. One-way ANOVA followed by Tukey's post hoc test.

Supplementary Table 1 The clinicopathological characteristics of patients across different cohorts.

Characteristics	Mannheim Cohort(n=98)	UROMOL(n=476)	Fudan Cohort(n=43)
**Age**			
Average(min-max)	69 (41-88)	69 (24-96)	67 (46-78)
**Gender**			
Female	19	109	11
Male	79	367	32
**Tissue Type**			
Paratumoral	0	0	0
Tumoral	98	476	43
**UCB Subtype**			
NMIBC	18	460	8
MIBC	80	16	35
**T Stage**			
Ta	1	345	1
Tis	6	3	0
T1	11	112	7
T2-T4	80	16	35
**N Stage**			
N0	69	0	41
Any N	25	0	2
Unknown	4	476	0

NMIBC: non muscle-invasive bladder cancer; MIBC: muscle-invasive bladder cancer.

Supplementary Table 2 Overview of the FFPE samples used for IHC and RNA FISH experiments.

Characteristics	IHC (n=43)	RNA FISH (n =4)
**Age**		
Average(min-max)	69 (48-89)	72 (68-78)
**Gender**		
Female	6	2
Male	37	2
**UCB Subtype**		
NMIBC	30	0
MIBC	13	4
**T Stage**		
Ta	15	0
Tis	15	0
T2-T4	13	4

NMIBC: non-muscle-invasive bladder cancer; MIBC: muscle-invasive bladder cancer.
